# Kinetic Patterns of Antibiotic Consumption in German Acute Care Hospitals from 2017 to 2023

**DOI:** 10.3390/antibiotics14030316

**Published:** 2025-03-18

**Authors:** Birgitta Schweickert, Niklas Willrich, Marcel Feig, Marc Schneider, Michael Behnke, Luis Alberto Peña Diaz, Christine Geffers, Imke Wieters, Karin Gröschner, Doreen Richter, Alexandra Hoffmann, Tim Eckmanns, Muna Abu Sin

**Affiliations:** 1 Healthcare-Associated Infections, Surveillance of Antimicrobial Resistance and Antimicrobial Consumption, Robert Koch Institute, 13353 Berlin, Germany; willrichn@rki.de (N.W.); richterd@rki.de (D.R.); eckmannst@rki.de (T.E.);; 2 Methods Development, Research Infrastructure and Information Technology, Koch-Institute, 13353 Berlin, Germany; feigm@rki.de (M.F.); schneiderm@rki.de (M.S.); 3 Institute for Hygiene and Environmental Medicine, Charité-Universitätsmedizin Berlin, Corporate Member of Freie Universität Berlin, Humbold-Universität zu Berlin and Berlin Institute for Health, 12203 Berlin, Germanyluis-alberto.pena-diaz@charite.de (L.A.P.D.);; 4 Federal Ministry of Health, 10117 Berlin, Germany

**Keywords:** antimicrobial consumption, surveillance, COVID-19-pandemic, antimicrobial stewardship, antimicrobial resistance

## Abstract

**Background:** Antimicrobial consumption (AMC) patterns, besides prescribing behaviors, reflect the changing epidemiology of infectious diseases. Routine surveillance data have been used to investigate the development of AMC from 2017 to 2023 and the impact of COVID-19 within the context of the framing time periods. **Methods:** Data from 112 hospitals, continuously participating from 2017 to 2023 in the national surveillance system of hospital antimicrobial consumption based at the Robert Koch Institute, were analyzed according to the WHO ATC (Anatomical Therapeutic Chemical)/DDD (Defined Daily Dose) method and categorized according to the WHO AWaRe-classification. AMC was quantified by consumption density (CD) expressed in DDD/100 patient days (PD) and DDD/100 admissions (AD). The time period was subdivided into three phases: pre-pandemic phase (2017–2019), main pandemic phase (2020–2021) and transition phase (2022–2023). Linear regression models have been used to determine the presence of an overall trend, the change in intra-phasic trends and phase-specific mean consumption levels over time. **Results:** From 2017 to 2023 total antibiotic consumption decreased by 7% from 57.1 to 52.9 DDD/100 PD. Four main kinetic patterns emerged across different antibiotic classes: Pattern 1 displays a decreasing pre-pandemic trend, which slowed down throughout the pandemic and transition phase and was exhibited by second-generation cephalosporins and fluoroquinolones. Pattern 2 reveals a rising pre-pandemic trend, which decelerated in the pandemic phase and accelerated again in the transition phase and was expressed by aminopenicillins/beta-lactamase inhibitors, beta-lactamase sensitive pencillins, azithromycin and first-generation cephalosporins. Pattern 3 shows elevated mean consumption levels in the pandemic phase exhibited by carbapenems, glycopeptides, linezolid and third-generation cephalosporins. Pattern 4 reveals a rising trend throughout the pre-pandemic and pandemic phase, which reversed in the transition phase without achieving pre-pandemic levels and was expressed by beta-lactamase resistant penicillins, daptomycin, fosfomycin (parenteral) and ceftazidime/avibactam. **Conclusions:** Kinetic consumption patterns across different antibiotic classes might reflect COVID-19-related effects and associated changes in the epidemiology of co-circulating pathogens and health care supply. Broad-spectrum antibiotics with persisting elevated consumption levels throughout the transition phase require special attention and focused antimicrobial stewardship activities.

## 1. Introduction

COVID-19 has posed serious challenges on health care provision in the hospital and outpatient sector [1]. The management of large numbers of patients suffering from a highly contagious disease with hitherto little information about clinical course and appropriate treatment options required large-scale implementation of rigorous infection prevention and control measures, substantial reorganization of hospital structures, development and ongoing adaptation of treatment guidelines [2,3]. The resulting implications for patient management, in particular changes in antibiotic prescribing practices, have been investigated by a large number of studies ranging from investigations in single centers or hospital units to multicenter-studies as well as to the analysis of data routinely collected in established national or supranational surveillance systems [4,5,6,7,8]. Systematic reviews and meta-analyses revealed that particularly in the first stage of the pandemic empirical antibiotic treatment was disproportionally high in relation to the detection-rate of bacterial co- and secondary infections [9,10,11,12,13]. Due to the growing scientific and empirical knowledge contributing to improved disease management, new treatment options and vaccinations, antibiotic consumption decreased during the further course of the pandemic [11,14,15,16,17,18,19,20]. Nevertheless, many studies reported persistently elevated levels of antibiotic consumption—particularly of broad-spectrum antibiotics, raising concerns about enhanced selection pressure for multidrug-resistant organisms and further spread of antimicrobial resistance [13,21,22,23]. 

Recently published data from German hospitals described an increase in total antibiotic consumption and selected broad-spectrum antibiotics from 2019 to 2022 [24]. In our study we used routine-surveillance data of the national Antimicrobial Consumption (AMC)-surveillance based at the Robert Koch Institute to investigate the development of hospital AMC from 2017 to 2023 with particular focus on the effects of the COVID-19 pandemic in consideration of the consumption trends presenting in the pre-pandemic phase (2017–2019) and the transitional phase (2022–2023), when the pandemic faded out and moved to an endemic state [25]. Characteristic kinetic consumption patterns were identified and interpreted in the context of the changing epidemiological situation.

## 2. Results

### 2.1. Hospitals

**Figure 1:**
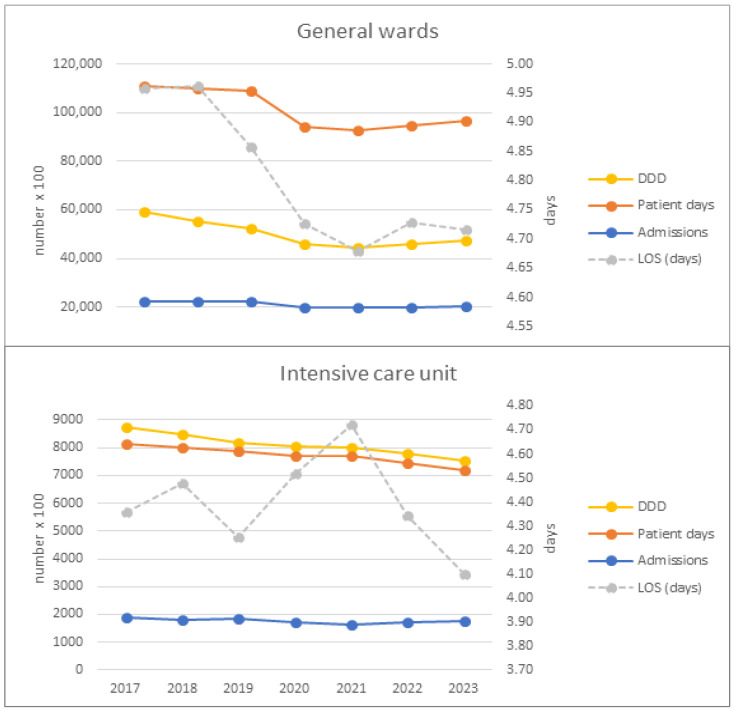
Temporal course of hospital activity parameters (admissions, patient days, length of stay (LOS)) and consumption volume (CV, DDD)) from 2017 to 2023 on general wards and ICUs.

The hospitals (n = 112), continuously participating in Antimicrobial Consumption (AMC)-surveillance, accounted for a total of 51.418 beds representing 12% of all general acute care hospital beds in Germany. The median bed count per hospital was 323 (IQR: 202–550) beds. Of the hospitals, 57% had a size of <400 beds, 64% were assigned to primary/secondary care hospitals and 36% to tertiary care hospitals. Hospital activity markers, namely patient days (PD), admissions (AD) and length of stay (LOS) and, concomitantly, the consumption volume (CV) decreased over the entire period by 13%, 9%, 6% and 19%, respectively. Subdivision into three phases and stratification by ward-type revealed that on general wards, the main decrease in PD, AD and CV occurred during the pandemic phase followed by a gradual increase in the transition phase without reaching pre-pandemic levels. On intensive care units (ICU), this pattern also applied to AD, whereas the other parameters exhibited a decrease throughout all phases. The course of LOS displayed diverging pandemic patterns characterized by a pandemic decrease in general wards and an increase in ICUs, which reversed in the transition phase (Figure 1).

### 2.2. Total Antibiotics and AWaRe-Categories

**Figure 2:**
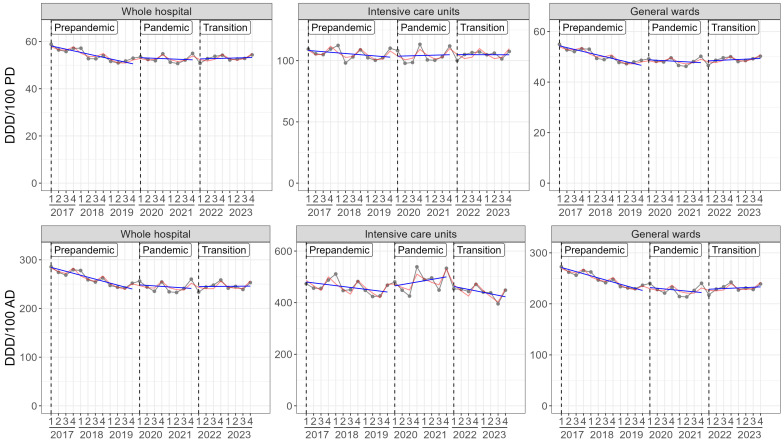
Kinetic patterns of total antibiotics displaying phase-specific trends of antibiotic consumption (DDD/100 patient days and DDD/100 admissions) according to three different phases: pre-pandemic phase (2017–2019), pandemic phase (2020–2021), transition phase (2022–2023). Observed consumption densities are shown in gray. Estimated regression models for the different phases are shown with (red) and without (blue) a term for seasonality (per quarter).

Data on total antibiotics and AWaRe-categories are displayed in Table 1, Table 2 and Table 3, Figure 2 and Tables S1–S3, Figure S1a–c. From 2017 to 2023 the overall consumption densities (CD) of antibiotics decreased from 57.1 to 52.9 DDD/100 PD by 7.2% (trend: *p* = 0.006) (Table 1). Considering the three phases separately revealed a change in intra-phasic trends. The decreasing trend presenting in the pre-pandemic phase slowed down in the pandemic phase displaying a slight and transient increase in the CDs in the first pandemic year, which reversed in the second pandemic year and was followed by a nearly stable course in the transition phase (Figure 2). Stratification by ward type revealed that this kinetic pattern was expressed predominantly by general wards resulting in a net overall decrease of 8.0% (trend: *p* = 0.003), whereas on ICUs only a marginal overall decrease of 2.2% (trend: *p* = 0.34) was observed. Calculating the consumption densities in DDD/100 AD revealed more marked differences: whereas the pre-pandemic decreasing trend on general wards merely slowed down in the pandemic phase (change of trend: *p* = 0.062), a significant rise by 11% (change of trend: *p* = 0.035) throughout both pandemic years occurred on ICUs and then reversed in the transition phase (Table 2, Figure 2).

Analyses based on the WHO AWaRe-categories revealed an overall rise in the CDs of the Access-group from 17.3 to 19.9 DDD/100 PD (trend: *p* < 0.001) accounting for 30% of total AMC in 2017 and 38% in 2023 (Table S1). Concomitantly, the Watch-group decreased from 36.2 to 28.5 DDD/100 PD (trend: *p* < 0.001) accounting for 63% of total antibiotics in 2017 and 54% in 2023. On a low level, the Reserve-group increased from 1.6 to 2.1 DDD/100 PD (trend: *p* < 0.001) accounting for 2.7% of total antibiotics in 2017 and 3.9% in 2023. As described for total antibiotics, the temporal courses of AD- and PD-related CDs showed that the most prominent differences occurred on ICUs during the pandemic phase (Table S2, Figure S1a–c).

### 2.3. Selected Antibiotic Classes

**Figure 3:**
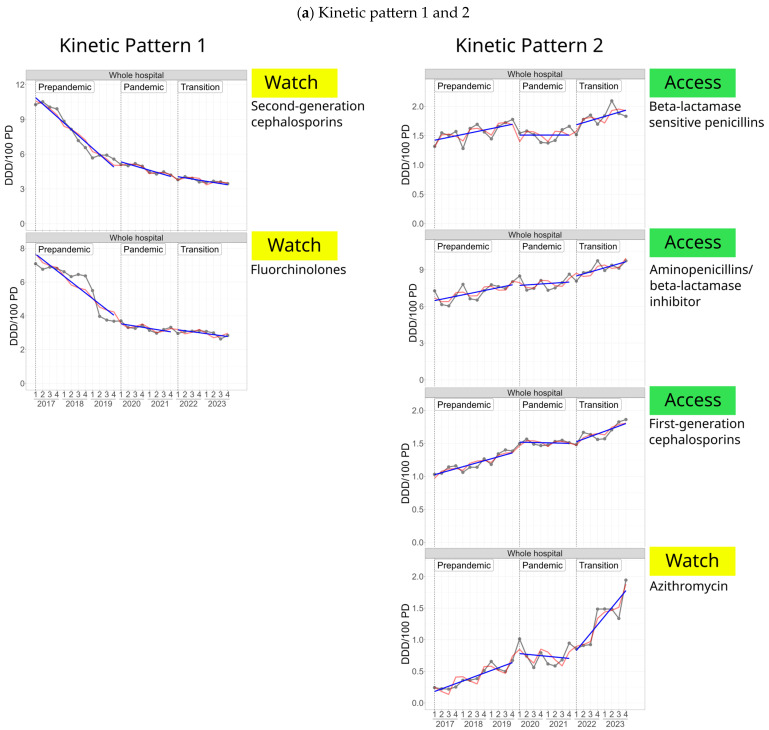

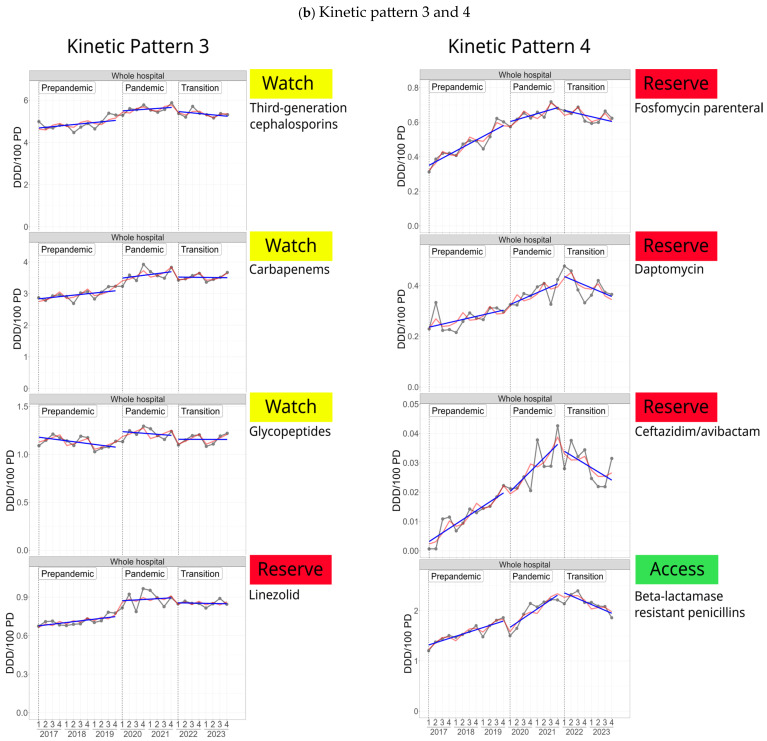
Kinetic patterns displaying phase-specific trends of antibiotic consumption (DDD/100 patient days) of selected antibiotic classes/substances according to three different phases: pre-pandemic phase (2017–2019), pandemic phase (2020–2021), transition phase (2022–2023); (**a**) Kinetic pattern 1 and 2 (**b**) Kinetic pattern 3 and 4. Observed consumption densities are shown in gray. Estimated regression models for the different phases are shown with (red) and without (blue) a term for seasonality (per quarter); antibiotic classes/substances have been allocated to the AWaRe-categories of WHO.

Data of selected antibiotic classes/substances expressed as DDD/100 PD are displayed in Figure 3 and in the Supplement Tables S4–S6 and Figure S2.

#### 2.3.1. Betalactams

From 2017 to 2023, the group of penicillins (J01C) increased continuously from 15.4 to 22.4 DDD/100 PD by 46% (trend, *p* < 0.001) accounting for 27% of total AMC in 2017 and 43% in 2023. An overall rise was observed across all major subgroups, thereby displaying different kinetic patterns; while aminopenicillins combined with beta-lactamase inhibitor (BLI), accounting for the most frequently used penicillin-subgroup, and beta-lactamase sensitive penicillins showed a pandemic slowdown of the previously increasing pre-pandemic trend, piperacillin/tazobactam and beta-lactamase resistant penicillins exhibited a strong pandemic increase, which in the transition phase further continued for piperacillin/tazobactam and partly reversed for beta-lactamase resistant penicillins. In contrast to penicillins, cephalosporins (J01DB/C/D/E) decreased from 16.2 to 10.7 DDD/100 PD by 34% (trend, *p* < 0.001) accounting for 28% of total AMC in 2017 and 20% in 2023. The strong overall decrease was mainly driven by second-generation cephalosporins (65%) depicting a steep decrease in the pre-pandemic phase which slowed down in the pandemic and transition phase. In contrast, first- and third-generation cephalosporins rose by 60% and 10%, respectively. While first-generation cephalosporins exhibited a kinetic pattern similar to aminopencillins/BLI and beta-lactamase sensitive penicillins showing a pandemic slowdown of an overall increasing trend, third-generation cephalosporins depicted a significant rise in the mean consumption level in the pandemic phase, which decreased again in the transition phase but without reaching pre-pandemic levels. As a result of the opposing trends, third-generation cephalosporins replaced second-generation cephalosporins as the most frequently used subgroup in 2023. The CDs of carbapenems (J01DH) increased from 2.9 to 3.5 DDD/100 PD by 21% (trend: *p* < 0.001). Besides piperacillin/tazobactam, carbapenems belonged to the most frequently used antibiotic class on ICUs (16%) with a seven times higher consumption level than on general wards. The temporal course showed a significant rise in the mean consumption level in the pandemic phase which remained constant in the transition phase.

#### 2.3.2. Macrolides

From 2017 to 2023 the CDs of macrolides (J01FA) decreased from 4.3 to 3.0 DDD/100 PD by 31% (trend: *p* < 0.001). The temporal course showed a declining trend in the pre-pandemic and pandemic phase, which partly reversed in the transition phase. The overall decrease was mainly caused by a strong decline of clarithromycin (70%), whereas on a lower level the consumption of azithromycin substantially increased (578%). Compared to 2017, azithromycin has replaced clarithromycin as the most frequently used macrolide in 2023. Despite the diverging temporal courses both substances showed peak consumption values in the first pandemic year.

#### 2.3.3. Fluoroquinolones

From 2017 to 2023 the CDs of fluoroquinolones decreased significantly from 6.9 to 2.9 DDD/100 PD by 58% (trend: *p* < 0.001). The main decrease occurred in the pre-pandemic phase, particularly from 2018 to 2019, slowed down in the pandemic phase and nearly leveled off in the transition phase. This kinetic pattern was equally expressed by ciprofloxacin, levofloxacin, moxifloxacin showing a decline by 66%, 39% and 57%, respectively. Moxifloxacin depicted a slightly different pattern with an isolated peak in 2020.

#### 2.3.4. Glycopeptides and Selected Reserve-Antibiotics

From 2017 to 2023, no overall increasing or decreasing trend was detected for glycopeptides. Nevertheless, a significant rise in the mean consumption level was observed in the pandemic phase, fully reversing in the transition phase. The substances constituting the group of Reserve-antibiotics (AWaRe-classification) showed a great diversity in terms of magnitude, temporal course of consumption and availability. Linezolid, daptomycin and fosfomycin (parenteral), accounting for 90% of the Reserve-group-consumption in 2023, showed an overall increase albeit to a different extent (23%, 31%, 63%, respectively). Other antibiotics, mainly for the treatment of multi-resistant Gram-negative pathogens, were rarely used and presented with varying kinetic patterns. While polymyxines (parenteral). showed an overall decreasing trend, ceftazidim/avibactam increased just as ceftolozan/tazobactam and on a lower level cefiderocol and aztreonam. The assessment over the whole time period was compromised for cefiderocol and ceftolozan/tazobactam, because cefiderocol was not available on the German market until 2020 and ceftolozan/tazobactam was not available in the year 2021. The CDs of tigecycline decreased between 2017 and 2023, with a transient pandemic rise in 2021.

#### 2.3.5. Common Kinetic Patterns of Selected Antibiotic Classes/Substances

Despite the temporal AMC-courses varied markedly, four main kinetic patterns, indicating an impact of the COVID-19 pandemic, emerged across different antibiotic classes (Figure 3a,b).

Kinetic pattern 1: The pattern displays a decreasing pre-pandemic trend, which slowed down throughout the pandemic and transition phase and was exhibited by second-generation cephalosporins and fluoroquinolones, both belonging to the Watch-group.

Kinetic pattern 2: The pattern displays a rising pre-pandemic trend, which slowed down in the pandemic phase and accelerated again in the transition phase and was expressed by aminopenicillins/BLI, beta-lactamase sensitive pencillins, azithromycin and first-generation cephalosporins.

Kinetic pattern 3: The pattern reveals elevated mean consumption levels in the pandemic phase, which to various degrees decreased in the transition phase. This pattern applies to carbapenems, glycopeptides, third-generation cephalosporins and linezolid.

Kinetic pattern 4: The pattern shows a rising trend throughout the pre-pandemic and pandemic phase, which reversed in the transition phase without returning to pre-pandemic levels and was expressed by beta-lactamase resistant penicillins, daptomycin, fosfomycin (parenteral) and ceftazidime/avibactam.

Apart from the three phases, clarithromycin, azithromycin and moxifloxacin showed an isolated consumption peak in the first quarter of the first pandemic year.

## 3. Discussion

Data derived from hospital-based routine-surveillance of Antimicrobial consumption (AMC) was used to investigate the development of antimicrobial consumption in 112 German acute care hospitals continuously providing data over a time period of seven years subdivided into three phases: the pre-pandemic phase (2017–2019), the main pandemic phase (2020–2021) and the transition phase (2022–2023) marking the transition from the pandemic to an endemic state. Given the heterogeneity of the temporal AMC-courses, we attempted to identify common kinetic consumption patterns across different antibiotic classes reflecting a potential impact of the COVID-19 pandemic.

The temporal course of total antibiotics reflects kinetic pattern 1 displaying a strong pre-pandemic decrease, which decelerated in the pandemic and transition phase. The slight spike in the first pandemic year was mainly caused by substances used for the treatment of respiratory tract infections (RTI) such as macrolides and moxifloxacin. Initial peak AMCs followed by a subsequent decrease have also been reported by other studies. [11,19,26,27,28]. Particularly in the first stage of the pandemic empirical antibiotic treatment was disproportionally high in relation to the detection-rate of bacterial co- and secondary infections [9,10,11,12,13]. A multi-center study conducted by the German competence center of community-acquired pneumonia (CAPNetz) found that 51% of COVID-19 patients received empirical antibiotic treatment [29]. This was explained by the initial uncertainties concerning disease management, which have successively been resolved due to growing scientific knowledge and clinical experience, which in turn informed ongoing adaptations of treatment guidelines [3,30]. This exemplarily applied to azithromycin presenting with a transient rise in the first pandemic year based on the erroneous assumption that the administration of azithromycin for different reasons might support the treatment of COVID-19 and which has since been disapproved [31]. In addition to updated treatment guidelines, vaccination as well as changes of the dominant COVID-19-variants associated with alleviated courses of COVID-19-disease contributed to a decline of AMC in the further course of the pandemic [20,32]. The overall decrease in total antibiotics by 7% found in our study does not necessarily contradict former results of a slight increase, as different time periods were considered [28]. The moderate changes suggest a limited impact of COVID-19 on antimicrobial use and consecutively on AMR. But, similar to other countries presenting with only marginal changes at the level of total antibiotics, concomitant COVID-19-related shifts predominantly of broad-spectrum-antibiotics such as piperacillin/tazobactam and third-generation cephalosporins indicated that the underlying antibiotic pattern has changed [16,17,24,33]. These effects might have contributed to the flattening of the pre-pandemic decreasing trend at the level of total antibiotics and the Watch-group observed in our study. For fluoroquinolones the main decrease occurred between 2018 and 2019 in response to the recommendations of the European Medicines Agency (EMA) for their restricted use [34]. The strong overall decrease in second generation cephalosporins can at least in part be attributed to Antimicrobial Stewardship (AMS)-activities, which discourage the use of cephalosporins as well as of fluoroquinolones in order to diminish unnecessary treatment with broad-spectrum antibiotics and the incidence of *Clostridioides difficile* (CDI)-infections [35]. This applies particularly to oral formulations of second-generation cephalosporins, because of their insufficient bioavailability. The observed decrease might be connected to a successive reduction in prolonged perioperative prophylaxis, which has been reported in the point prevalence survey (PPS) on hospital acquired infection and antimicrobial use 2022–2023 coordinated by ECDC [36]. Concurrently, a significant decrease in the prevalence of CDI has been described. In the transition phase, the decrease in both antibiotic classes leveled off, possibly signaling that the limit of the reduction potential was near to be achieved or that additional AMS-efforts were required. Considering the overall trend of betalactams and fluoroquinolones from 2017 to 2023, it becomes evident, that irrespective of the modifying effects of the pandemic, a continuous shift from the use of cephalosporins and fluoroquinolones to an increased use of the group of penicillins took place, which was also confirmed by other studies [24,36].

Overall, the pandemic changes on ICUs were less marked than on general wards. However, in contrast to PD-related CDs, AD-related CDs showed a significant pandemic rise in the mean consumption levels on ICUs, which subsequently reversed in the transition phase. This was connected with an increased length of stay in the pandemic phase probably due to the need for prolonged ventilation of COVID-19-patients consecutively associated with a higher incidence of nosocomial infections [13,37]. Accordingly, the higher AD-related CD-levels of the pandemic phase could be an expression of longer treatment durations and/or of successive treatments of the single patient. Similar results have been found by other studies reinforcing the recommendation to use more than one metric to provide a more complete picture of AMC particularly in changing external circumstances affecting disease epidemiology, health care provision and health care-seeking behaviors [38,39].

Kinetic pattern 2, indicating an influence of COVID-19, displays an increasing pre-pandemic trend, which slowed down in the pandemic phase and accelerated again in the transition phase. This constellation might reflect changes in disease patterns and/or pathogen spectrum. Due to COVID-19 containment measures, the incidence of RTI caused by co-circulating viruses and associated bacterial pathogens such as *Streptococcus pneumoniae* and *Haemophilus influenzae* decreased significantly, which corresponds closely to the pandemic slowdown of the increasing pre-pandemic trend of aminopenicillins/BLI, beta-lactamase sensitive penicillins and azithromycin seen in our study [40]. Subsequently, the epidemiological situation reversed presenting with a strong resurgence of RTI in the winter season 2022/23 [41,42]. Particularly an increase in scarlet fever and invasive *Streptococcus pyogenes*-infections was reported from Germany and other countries [43,44]. This probably resulted in a renewed acceleration of antibiotic consumption in the transition phase. In fact, the demand—especially of the liquid forms of the above-mentioned agents—exceeded the existing resources leading to transient supply shortages [45]. The resurgence of RTIs has been explained by the suspension of COVID-19-related containment measures accompanied by an elevated susceptibility of the population [43]. The increase in azithromycin could additionally be attributed to an update of the German Community Acquired Pneumonia (CAP)-guideline in 2021 favoring the use of azithromycin over clarithromycin [46]. The observed overall increase in first-generation cephalosporins, also sharing kinetic pattern 2, might, at least in part, be due to a compensation for the declining use of second-generation cephalosporins, whereas the pandemic slowdown probably goes back to a reduction in elective surgery and a consecutively reduced need for perioperative prophylaxis [2].

Kinetic pattern 3, indicating a potential influence of COVID-19, primarily applied to third-generation cephalosporins, carbapenems, glycopeptides and linezolid. It is characterized by a significant upward shift of the mean consumption level in the pandemic phase compared to the pre-pandemic phase. Particularly for third-generation cephalosporins, but also for the other mentioned substances, an increased consumption during the pandemic, exceeding the expected values, has been reported by many other studies [11,19,26,38]. Kinetic pattern 4, expressed by beta-lactamase resistant penicillins, daptomycin, fosfomycin (parenteral) and ceftazidime/avibactam, shows a continuous rise throughout the pre-pandemic and pandemic phase, which partially reversed in the transition phase. Common to both kinetic patterns is that the observed decline in the transition phase, underscoring an assumed COVID-19-related effect, was not sufficient to achieve pre-pandemic consumption levels. The rise in ceftazidime/avibactam was probably partly due to a successive replacement of polymyxins, which simultaneously decreased significantly. Nevertheless, its increased use together with cefiderocol and aztreonam, particularly in 2022, might be a response to a rise in NDM-1-producing *Klebsiella peumoniae* associated with the refugee movement from Ukraine [47]. This is in line with data of the rising incidence in carbapenem-resistant *Klebsiella pneumoniae* reported by other surveillance systems [48]. However, the very low basic level of use indicates that these substances generally were prudently used in German hospitals. The pandemic increase in beta-lactamase-resistant penicillins and of the above-mentioned broad-spectrum antibiotics may indicate an increase in nosocomial infections associated with the longer stay of critically ill COVID-19-patients and the use of immunosuppressive agents such as corticosteroids recommended in COVID-19 treatment guidelines [11,14,30,49]. *Staphylococcus aureus* (*S. aureus*) was reported to be among the most frequently detected pathogens causing secondary infections in COVID-19 [50]. But, irrespective of any potential pandemic effect, the overall increasing trend of beta-lactamase resistant penicillins might in part be due to AMS-efforts aiming to optimize the management of *S.* aureus-BSIs, e.g., concerning the dose and length of therapy [30]. Furthermore, a recently published German study reported an increase in *S. aureus*-BSI in tertiary care hospitals over a time span of 14 years [51]. Another reason for the strong rise in the use of betalactamase-resistant penicillins might be an increase in Methicillin-sensitive *S. aureus* (MSSA)-positive BSI at the cost of Methicillin-resistant *S. aureus* (MRSA)-positive BSI, which has been described in a study analyzing the data of *S. aureus*-BSI collected in the framework of EARS-Net [52].

The sustained elevated levels of broad-spectrum antibiotics beyond the first stages of the pandemic observed in our study, have also been reported by other authors [13,15,53]. The clarification of the underlying reasons is complex. According to EARS-Net-data, an increasing trend of German resistance-rates over the last 5 years could not be detected, except for carbapenem-resistant *Klebsiella pneumoniae* already discussed above [48]. In contrast, a continued decrease was observed for MRSA- and VRE-rates. In addition, the PPS did not reveal an increase in the prevalence of the hospital acquired infections between 2016 and 2022 [36]. On the other hand, the renewed surge of RTI in the winter season 2022/23 and consecutive high hospitalization rates—particularly of the elderly—as well as changes in health care supply, might have contributed to a higher share of patients suffering from severe conditions [42,54]. This assumption might indirectly be supported by the persisting low level of hospital activity parameters observed in our data and confirmed by recent nationwide statistics [55]. Another potential reason for the presumed change in patient mix compared to the pre-pandemic phase pertains to a significant shift in patients with so called “ambulatory care sensitive conditions” from hospital to outpatient care [56]. Another noteworthy factor possibly contributing to continued elevated CD-levels during the transition phase might go back to the amendments of EUCAST clinical breakpoints with the introduction of the new intermediate (I) [57]. A Swiss study found that the new “I” was associated with the application of higher doses of piperacillin/tazobactam and an increased use of carbapenems [58]. In this context, a survey among German clinicians revealed a lack of knowledge about the changes and their consequences for treatment and highlighted the need for targeted AMS-activities [59].

The study has several strengths and limitations. One strength is the length of the observed time period, which allows to assess the impact of COVID-19 on AMC in the context of consumption-trends before and beyond the main pandemic phase. This setting also made it possible to identify overall AMC-trends beyond the COVID-19-effects. Another strength is the multicentered approach presenting with a large sample of hospitals continuously participating in routine surveillance over the whole time period. A disadvantage is the voluntary nature of participation resulting in a convenience sample with limitations in national and regional representativeness. Thus, in our study, hospitals of >400 beds were overrepresented compared to the respective share of this size category in the entire German hospital landscape (37% versus 24%). Together with the fact that the burden of severely ill COVID-19 patients was higher in larger hospitals, the magnitude and pattern of AMC might be biased towards higher AMC-levels [60]. Furthermore, a selection bias might have been introduced, if hospitals, continuously participating in a voluntary surveillance system, might differ from non-participating hospitals, e.g., with respect to their engagement in AMS-activities [61]. Another limitation is that data collection was based on aggregated data, which does not allow the assessment of individual determinants of antibiotic prescribing such as indication. In addition, the data were collected in the framework of routine AMC-surveillance which did not include concomitant information, e.g., about the incidence of hospital-acquired infections, case mix index, antimicrobial resistance rates, drug shortages or about the varying degrees of AMS-implementation facilitating interpretation of the data. Furthermore, the subdivision of the observed time period in the three phases, as well as the categorization in common kinetic patterns, may not necessarily fit the temporal course of every single antibiotic adequately. Nevertheless, the intended simplification might contribute to a better understanding of the complex, interacting AMC-patterns of a wider spectrum of antibiotics, instead of focusing only on the most prominent ones.

In summary, apart from the slight overall decrease in total AMC, the temporal courses of selected antibiotic classes/substances revealed a complex pattern reflecting the impact of the COVID-19-pandemic and associated epidemiological effects on clinical care and hospital organization. Besides a potential overuse of antibiotics, particularly in the early stages of the pandemic, changes in patient mix, pathogen-spectrum and hospital care supply determined the temporal development of AMC-patterns. The continuing increase or persisting elevated levels of selected broad-spectrum- and reserve-antibiotics need to be closely monitored and continuously evaluated in the context of the epidemiological situation and the determinants of hospital management. Particularly in such dynamic situations, it is of great value to have well-established AMS structures equipped with adequate personnel resources in place in order to assure a constantly high quality of antibiotic management. The consolidation and strengthening of AMS and broad access to infectious disease expertise need to be important considerations when preparing for future pandemics and health emergencies.

## 4. Materials and Methods

Data from 112 acute care hospitals, continuously participating in the national Antimicrobial Consumption (AMC)-surveillance system from 2017 to 2023, were analyzed according to the ATC (Anatomical Therapeutic Chemical)/DDD (Defined Daily Dose)-method of WHO. Details of the surveillance system have been described elsewhere [62]. AMC was quantified by consumption density (CD) expressed in DDD/100 patient days (PD) and DDD/100 admissions (AD). In addition, the temporal development of basic parameters comprising PD, AD, length of stay (LOS) and consumption volume (CV, DDD) were considered. The observed time period has been subdivided into three phases: pre-pandemic phase (2017–2019), pandemic phase (2020–2021) and transition phase (2022–2023), when the pandemic faded out and moved to an endemic state. In order to obtain a preferably homogenous sample of hospitals, pediatric, psychiatric and rehabilitation departments have been excluded from analysis. Data on total antibiotics (A07AA, J01, J04AB02, P01AB01) and the WHO AWaRe-categories [63] have been analyzed at hospital level and stratified by ward type (intensive care unit (ICU)/general ward). Characteristic consumption patterns across different antibiotic classes were identified.

### Statistical Analysis

Data have been analyzed according to three objectives:

1. Investigation of the presence of a linear increasing or decreasing trend or no linear trend in AMC over the entire time period (2017–2023)
2. Investigation of differences in phase-specific mean consumption levels
3. Investigation of the presence and change in intra-phasic trends according to the three phases

As a base model (model 1) we considered a linear regression with quarterly aggregated antibiotic consumption as the outcome using the current quarter (1, 2, 3, 4) as a predictor to model the seasonal component of the consumption patterns and a continuous linear time trend as a predictor for overall changes across the study period. Two additional variants of the model were considered: A second model (model 2) with the pandemic phase added as a categorical predictor—allowing for different predicted base line levels of consumption in the different phases of the pandemic—and a third model (model 3) adding an interaction term for the continuous time variable and pandemic phase which allowed to estimate separate linear time trends for each phase of the pandemic.

The presence and magnitude of a possible linear time trend in the model 1 was used to assess objective 1 stated above. F-tests, performed for comparison of model 1 and model 2, were used to assess objective 2. Comparisons between model 2 and model 3 with F-Tests were used in order to investigate the presence of phase-specific trends as formulated in objective 3. *p*-values < 0.05 were considered statistically significant. All analyses were performed using R (version 4.2.1, R Foundation for Statistical Computing, Vienna, Austria).

## Figures and Tables

**Table 1:** Trends of antimicrobial consumption of total antibiotics from 2017 to 2023 expressed in DDD/100 patient days and DDD/100 admissions and stratified by ward type.

		Pre-Pandemic			Pandemic		Transition					
		2017	2018	2019	2020	2021	2022	2023	Difference 2017–2023	Change (%)	Trend ^a^ (95%CI)	Trend *p*-Value
Whole hospital	PD ^b^	57.1	54.2	51.8	53.1	52.3	52.9	53.0	−4.1	−7.2	−0.13 (−0.22; −0.04)	0.006
	AD ^b^	277.1	263.7	246.1	247.5	242.2	246.0	244.7	−32.4	−11.7	−1.21 (−1.71; −0.72)	<0.001
												
ICU ^c^	PD	107.3	105.7	103.7	104.5	104.0	104.6	105.0	−2.4	−2.2	−0.08 (−0.25; 0.09)	0.344
	AD	467.9	472.9	441.1	472.2	490.8	454.6	430.4	−37.5	−8.0	−0.87 (−2.12; 0.38)	0.162
												
General Ward	PD	53.3	50.4	47.9	48.7	47.8	48.7	49.0	−4.2	−8.0	−1.30 (−1.85; −0.75)	<0.001
	AD	264.1	249.8	232.6	230.4	223.7	230.5	231.2	−32.9	−12.5	−0.14 (−0.23; −0.05)	0.003
												

^a^ Time trend can be interpreted as expected change in consumption between consecutive quarters. Values displayed represent point estimates, 95% CIs and *p*-values; ^b^ PD: DDD/100 patient days; AD: DDD/100 admissions; ^c^ ICU, Intensive Care Unit.

**Table 2:** Antimicrobial consumption (DDD/100 patient days and DDD/100 admissions) of total antibiotics from 2017 to 2023 subdivided into three phases: pre-pandemic phase (2017–2019), pandemic phase (2020–2021), transition phase (2022–2023): intra-phasic trends and inter-phasic changes in trend (pre-pandemic–pandemic and pandemic–transition).

		Pre-Pandemic Phase				Pandemic Phase				Transition Phase			
		2017–2019				2020–2021				2022–2023			
		Diff ^a^ 17–19	Change (%)	Trend	*p*-Value	Diff 19−21	Change (%)	Change of Trend ^b^	*p*-Value	Diff 21–23	Change (%)	Change of Trend ^c^	*p*-Value
Whole hospital	PD ^d^	−5.3	−9.2	−0.69 (−0.88; −0.50)	<0.001	0.5	1.0	0.56 (1.03; 0.09)	0.019	0.7	1.3	0.21 (0.80; −0.37)	0.630
	AD ^d^	−31	−11.2	−4.05 (−5.16; −2.94)	<0.001	−3.9	−1.6	3.02 (5.80; 0.23)	0.032	2.6	1.1	1.19 (4.62; −2.23)	0.656
													
ICU ^e^	PD	−3.6	−3.4	−0.50 (−1.13; 0.14)	0.119	0.2	0.2	0.66 (2.25; −0.93)	0.550	1.0	1.0	−0.18 (1.77; −2.14)	0.970
	AD	−26.8	−5.7	−3.54 (−6.71; −0.37)	0.031	49.7	11.3	8.51 (16.46; 0.57)	0.035	−60.4	−12.3	−10.68 (−0.90; −20.47)	0.031
													
General Ward	PD	−31.4	−11.9	−0.70 (−0.88; −0.53)	<0.001	−8.9	−3.8	0.54 (0.98; 0.10)	0.014	7.4	3.3	0.30 (0.84; −0.25)	0.368
	AD	−5.4	−10.1	−4.11 (−5.25; −2.97)	<0.001	−0.1	−0.2	2.72 (5.57; −0.13)	0.062	1.2	2.5	2.05 (5.55; −1.46)	0.321
													

^a^ Diff: difference in DDD/100 PD and DDD/100/AD; ^b^ change in trend from the pre-pandemic to the pandemic phase; ^c^ change in trend from the pandemic to the transition phase; ^d^ PD: DDD/100 patient days; AD: DDD/100 admissions; ^e^ ICU, Intensive Care Unit.

**Table 3:** Mean consumption levels (DDD/100 patient days and DDD/100 admissions) of total antibiotics: differences between the pre-pandemic (2017–2019) and the pandemic phase (2020–2021) and the pandemic and the transitional phase (2022–2023).

		Pre-Pandemic Phase	Pandemic Phase	Transition Phase	Difference		Difference	
		2017–2019	2020–2021	2022–2023	Pre-Pandemic—Pandemic		Pandemic—Transition	
		Mean Value	Mean Value	Mean Value	Difference		Difference	
		(95%CI)	(95%CI)	(95%CI)	(95%CI)	*p*-Value	(95%CI)	*p*-Value
Whole hospital	PD ^a^	54.3 (53.7; 55.0)	52.7 (51.9; 53.5)	52.9 (52.2; 53.7)	−1.6 (−0.62; −2.63)	0.003	0.2 (1.32; −0.87)	0.673
	AD ^a^	262.2 (258.5; 265.9)	244.8 (240.2; 249.4)	245.4 (240.8; 250.0)	−17.42 (−11.52; −23.32)	<0.001	0.60 (7.07; −5.86)	0.847
								
ICU ^b^	PD	105.5 (103.4; 107.7)	104.2 (101.6; 106.8)	104.8 (102.2; 107.4)	−1.34 (2.03; −4.71)	0.415	0.59 (4.28; −3.10)	0.742
	AD	460.5 (449.9; 471.2)	482.4 (469.3; 495.4)	442.6 (429.5; 455.6)	21.83 (38.69; 4.97)	0.014	−39.77 (−21.30; −58.24)	<0.001
								
General Ward	PD	50.5 (49.9; 51.1)	48.3 (47.5; 49.0)	48.9 (48.2; 49.6)	−2.23 (−1.29; −3.16)	<0.001	0.62 (1.64; −0.41)	0.222
	AD	248.7 (244.9; 252.6)	227.0 (222.3; 231.6)	230.9 (226.2; 235.5)	−21.78 (−15.74; −27.82)	<0.001	3.90 (10.51; −2.71)	0.202
								

^a^ PD: DDD/100 patient days; AD: DDD/100 admissions; ^b^ ICU, Intensive Care Unit.

## Data Availability

The original contributions presented in this study are included in the article/Supplementary Materials. Further inquiries can be directed to the corresponding author.

## Supplementary Materials

The following supporting information can be downloaded at: https://www.mdpi.com/article/10.3390/antibiotics14030316/s1, Table S1: Trends of antimicrobial consumption (DDD/100 patient days and DDD/100 admissions) of the AWaRe-categories (WHO) from 2017 to 2023; Table S2: Antimicrobial consumption (DDD/100 patient days and DDD/100 admissions) of the AWaRe-categories (WHO) from 2017 to 2023 subdivided into pre-pandemic phase (2017–2019), pandemic phase (2020–2021), transition phase (2022–2023): display of intra-phasic trends and inter-phasic changes in trend (pre-pandemic—pandemic and pandemic—transition); Table S3: Mean consumption levels (DDD/100 patient days and DDD/100 admissions) of the AWaRe-categories (WHO): difference between the pre-pandemic (2017–2019) and the pandemic phase (2020–2021) and between the pandemic and the transitional phase (2022–2023); Table S4: Trends of antimicrobial consumption (DDD/100 patient days) of selected antibiotic classes/substances from 2017 to 2023; Table S5: Antimicrobial consumption (DDD/100 patient days) of selected antibiotics from 2017 to 2023 subdivided into pre-pandemic phase (2017–2019), pandemic phase (2020–2021), transition phase (2022–2023): display of intra-phasic trends and inter-phasic changes in trend (pre-pandemic—pandemic and pandemic—transition); Table S6: Mean consumption levels (DDD/100 patient days) of selected antibiotics: difference between the pre-pandemic (2017–2019) and the pandemic phase (2020–2021) and between the pandemic and the transitional phase (2022–2023); Figure S1: Kinetic patterns of AWaRe-categories displaying phase-specific trends of antibiotic consumption (DDD/100 patient days and DDD/100 admissions) according to three different phases: pre-pandemic phase (2017–2019), pandemic phase (2020–2021), transition phase (2022–2023); (a) Access-group; (b) Watch-group; (c) Reserve-group; Figure S2: Kinetic patterns of selected antibiotic classes/substances displaying phase-specific trends of antibiotic consumption (DDD/100 patient days) according to three different phases: pre-pandemic phase (2017–2019), pandemic phase (2020–2021), transition phase (2022–2023); (a) Penicillins; (b) Cephalosporins; (c) Macrolides; (d) Fluoroquinolones; (e) Selected antibiotic classes/substances.

## Abbreviations

The following abbreviations are used in this manuscript:

MDPI	Multidisciplinary Digital Publishing Institute
DOAJ	Directory of open access journals
TLA	Three letter acronym
LD	Linear dichroism
