# Supplementary material for: Kinetic Patterns of Antibiotic Consumption in German Acute Care Hospitals from 2017 to 2023
Source: Antibiotics (Basel). 2025 Mar 18;14(3):316. doi: 10.3390/antibiotics14030316 (PMC11939389; doi:10.3390/antibiotics14030316)
Supplement: Supplementary file 1 [file antibiotics-14-00316-s001.zip › Supplement Figure S1a-c.docx]

**Supplement Figure S1a-c. Kinetic patterns of the AWaRe-categories displaying phase-specific trends of antibiotic consumption (DDD/100 patient days and DDD/100 admissions) according to three different phases: pre-pandemic phase (2017-2019), pandemic phase (2020-2021), transition phase (2022-2023); (a)** Access-group; **(b)** Watch-group; **(c)** Reserve-group

1. **Access group**^a^


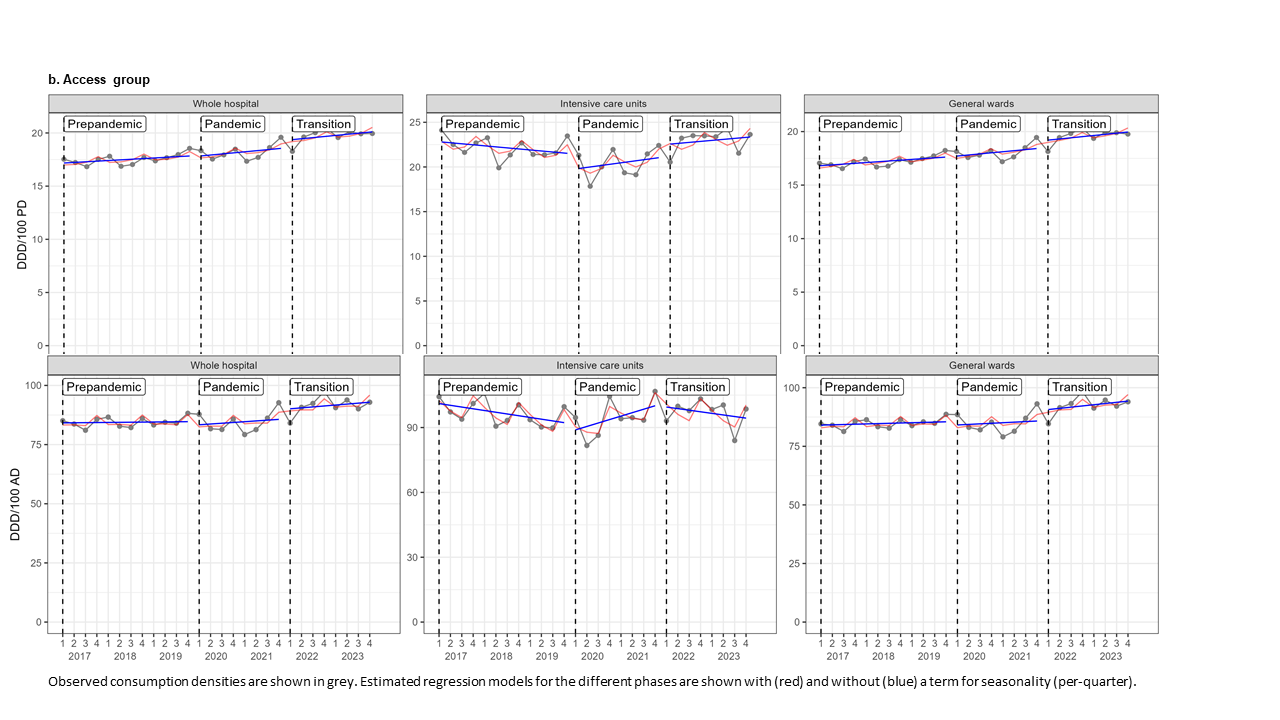


1. **Watch group**

**
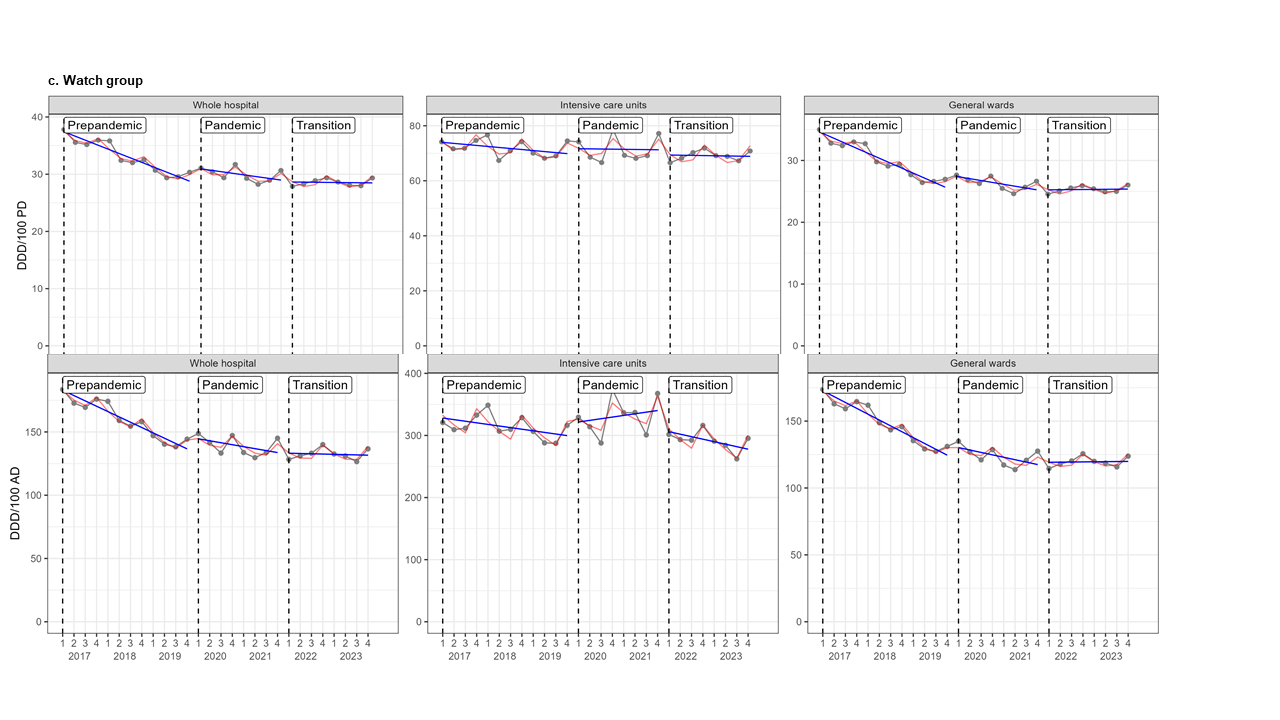
**

1. **Reserve Group**

**
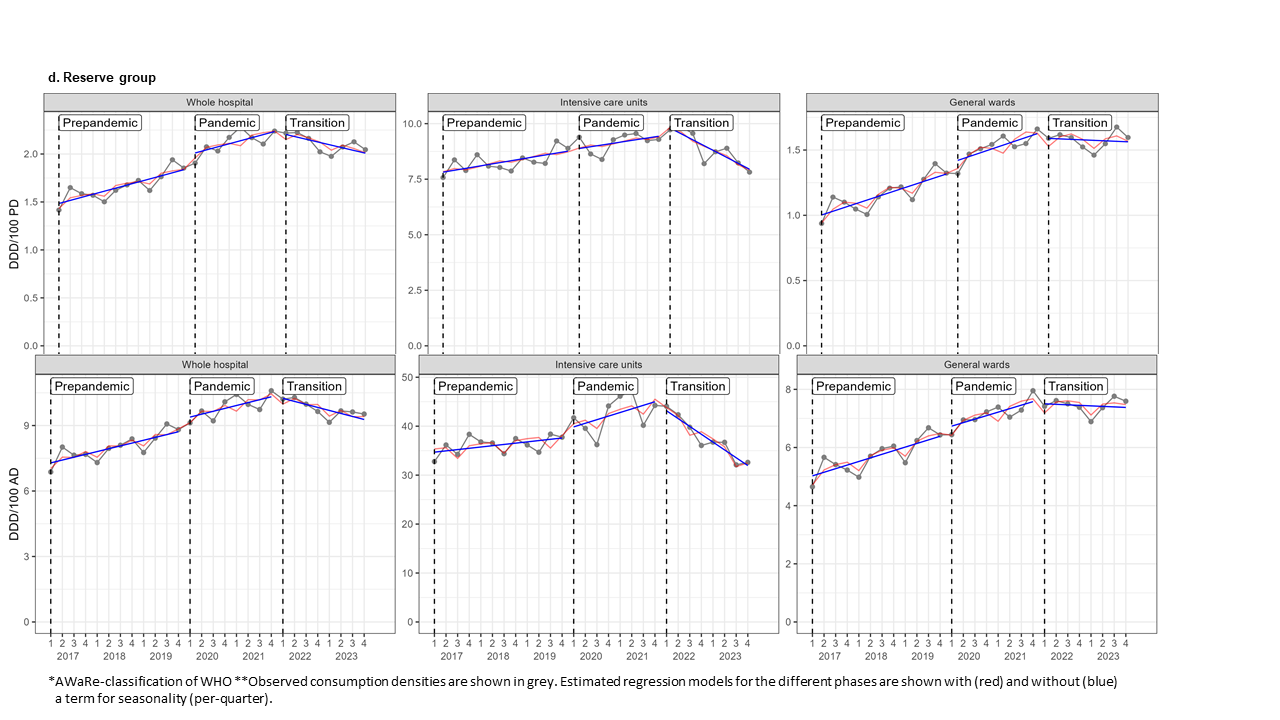
**

^a^Observed consumption densities are shown in grey. Estimated regression models for the different phases are shown with (red) and without (blue) a term for seasonality (per-quarter)
