# Supplementary material for: Kinetic Patterns of Antibiotic Consumption in German Acute Care Hospitals from 2017 to 2023
Source: Antibiotics (Basel). 2025 Mar 18;14(3):316. doi: 10.3390/antibiotics14030316 (PMC11939389; doi:10.3390/antibiotics14030316)

**Supplement Figure S2. Kinetic patterns of selected antibiotic classes/substances displaying phase-specific trends of antibiotic consumption (DDD/100 patient days) according to three different phases: pre-pandemic phase (2017-2019), pandemic phase (2020-2021), transition phase (2022-2023); (a)** Penicillins; **(b)** Cephalosporins; **(c)** Macrolides; **(d)** Fluoroquinolones; **(e)** Selected antibiotic classes/substances. Observed consumption densities are shown in grey. Estimated regression models for the different phases are shown with (red) and without (blue) a term for seasonality (per-quarter).

1. **Penicillins**


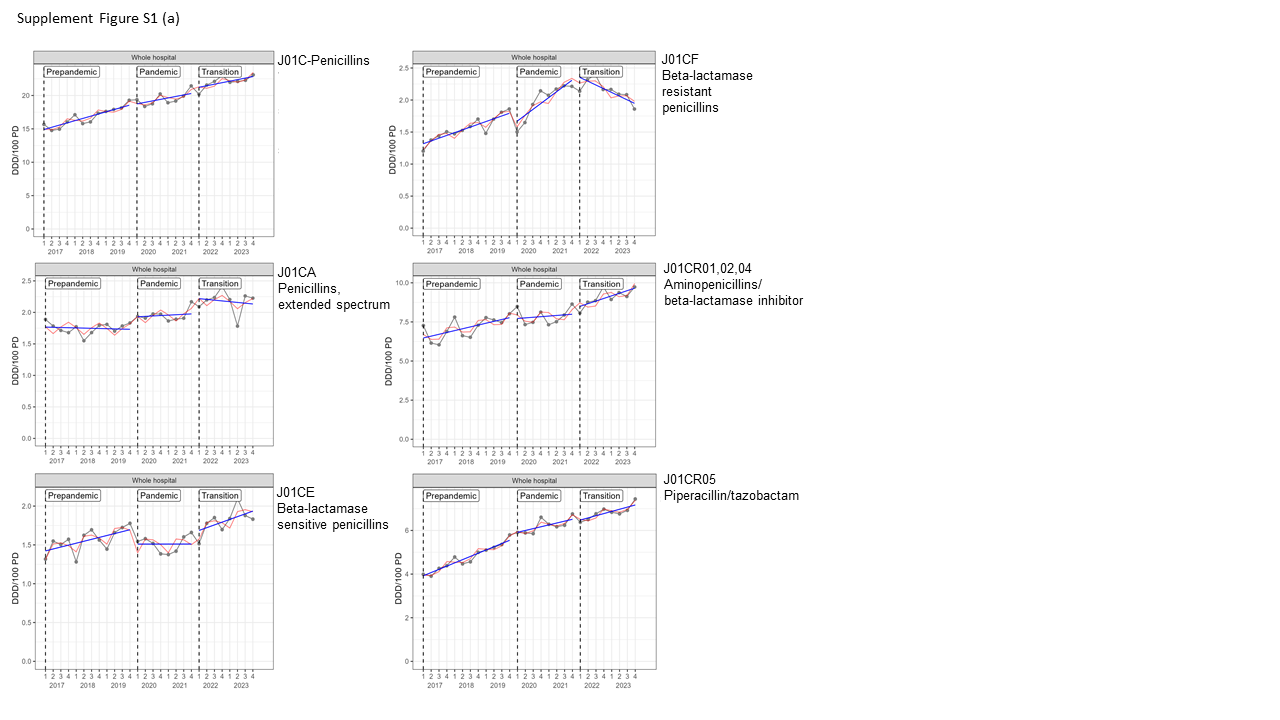


1. **Cephalosporins**


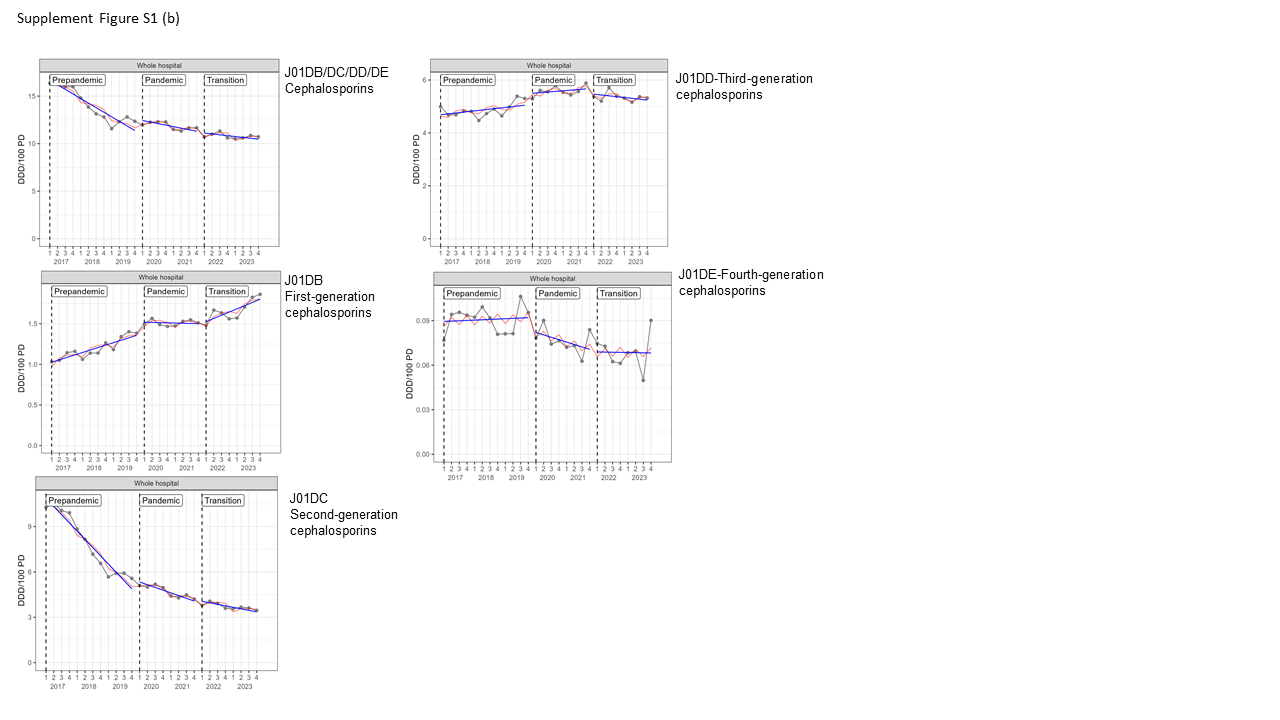


1. **Macrolides**


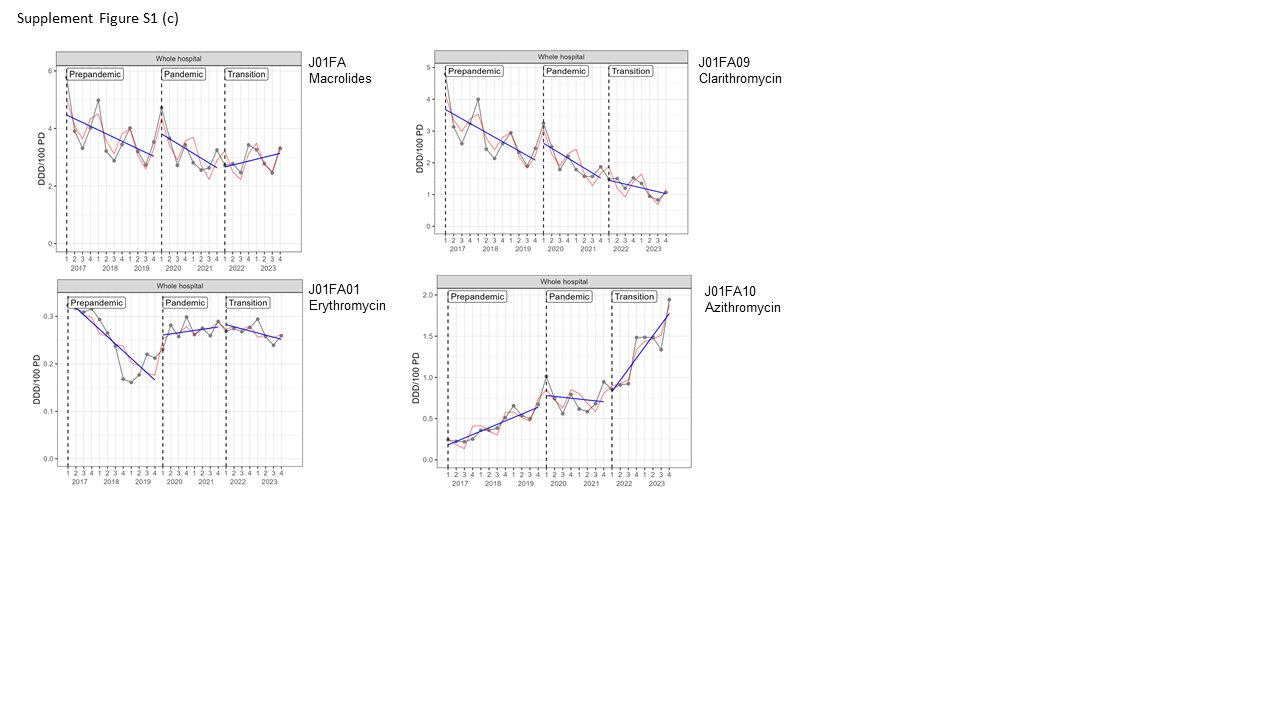


1. **Fluoroquinolones**


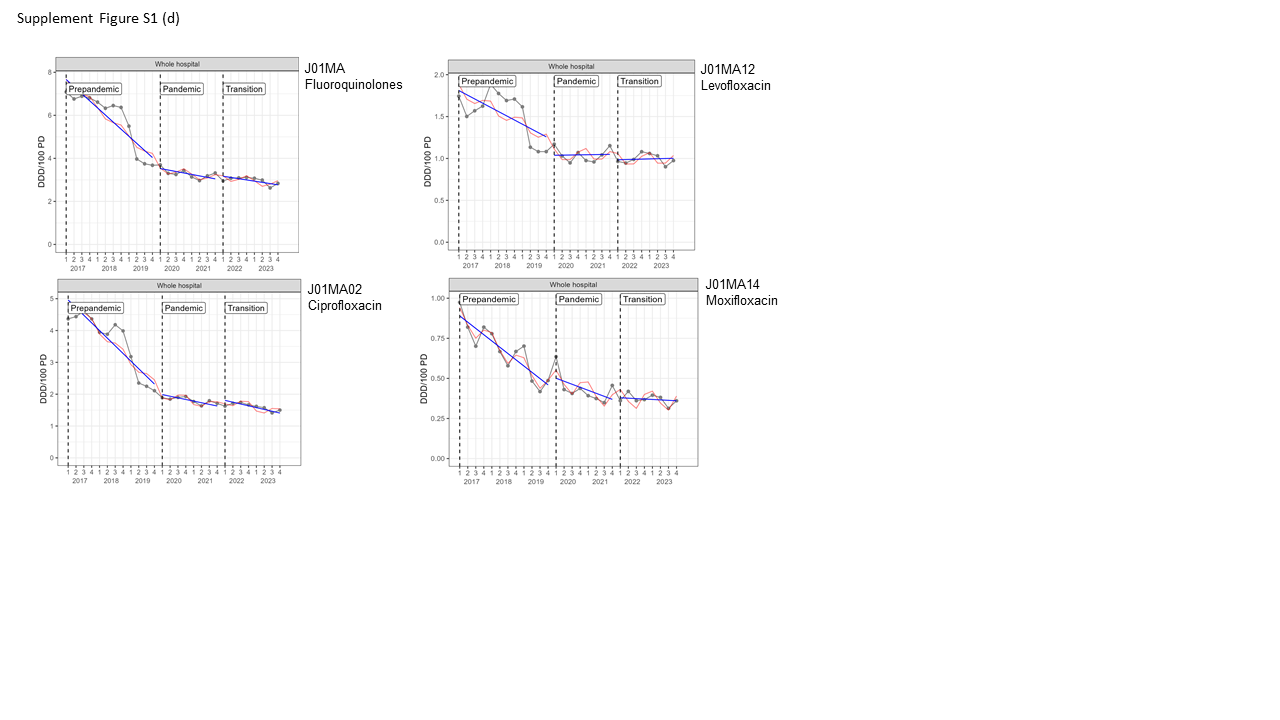


1. **Selected antibiotic classes/substances**


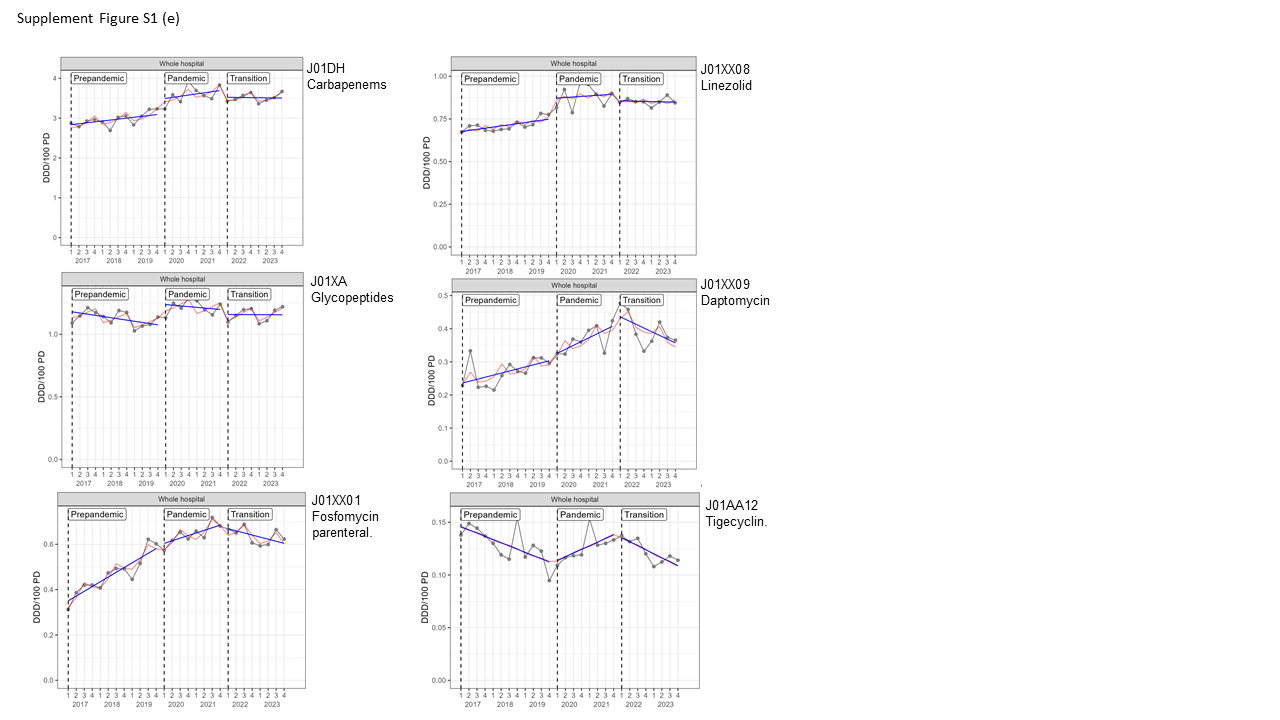


**Selected antibiotic classes/substances (continued)**


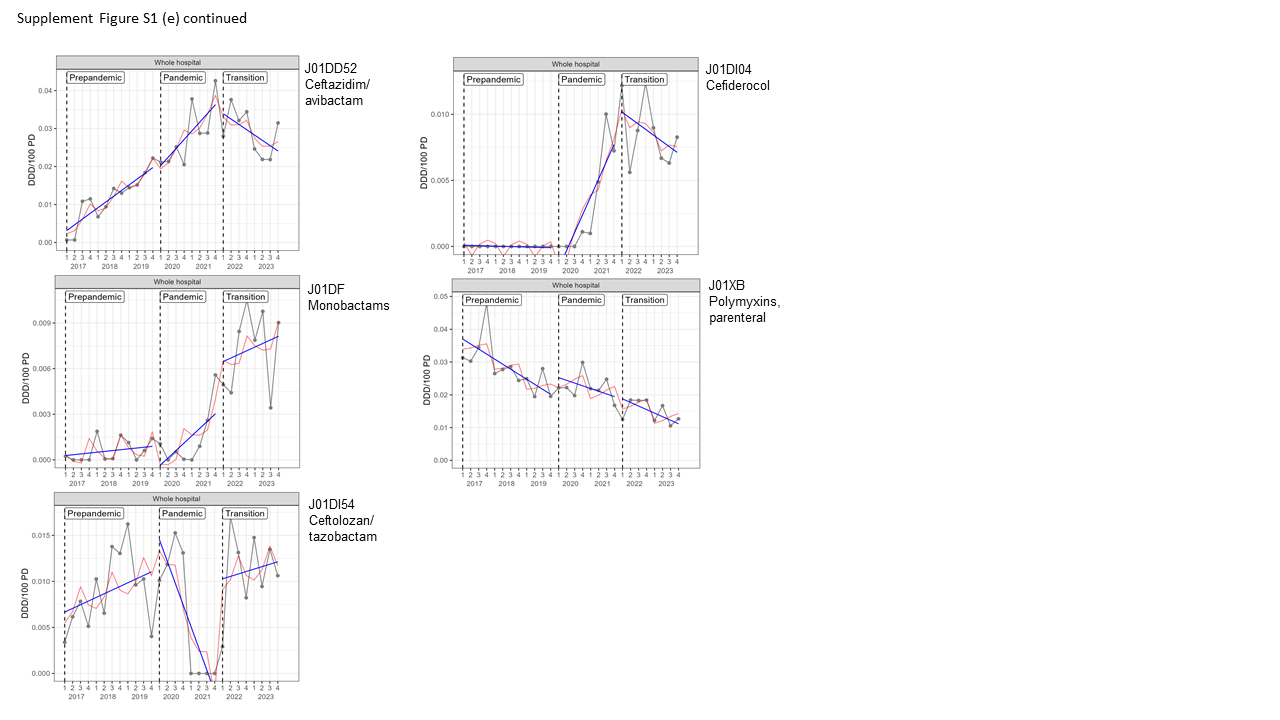

Supplement: Supplementary file 1 [file antibiotics-14-00316-s001.zip › Supplement Figure S2.docx]
