# Supplementary material for: Kinetic Patterns of Antibiotic Consumption in German Acute Care Hospitals from 2017 to 2023
Source: Antibiotics (Basel). 2025 Mar 18;14(3):316. doi: 10.3390/antibiotics14030316 (PMC11939389; doi:10.3390/antibiotics14030316)
Supplement: Supplementary file 1 [file antibiotics-14-00316-s001.zip › Supplement Table S1.docx]

**Supplement Table S1. Trends of antimicrobial consumption of the AWaRe-categories (WHO) from 2017 to 2023 expressed in DDD/100 patient days and DDD/100 admissions and stratified by ward type.**

|  | |  | | **Pre-pandemic** | | | | | | **Pandemic** | | | | **Transition** | | | |  | | | | | | | |  | | | | | |
| --- | --- | --- | --- | --- | --- | --- | --- | --- | --- | --- | --- | --- | --- | --- | --- | --- | --- | --- | --- | --- | --- | --- | --- | --- | --- | --- | --- | --- | --- | --- | --- |
|  | |  | | **2017** | | **2018** | | **2019** | | **2020** | | **2021** | | **2022** | | **2023** | | | |  | | **Difference**  **2017-23** | | **change (%)** | | | | Trend^a^ **(95%CI)** | | **Trend p-value** | |
| **Access-antibiotics** | |  | |  | |  | |  | |  | |  | |  | |  | | | |  | |  | |  | | | |  | |  | |
| Whole hospital | | PD ^b^ | | 17.3 | | 17.4 | | 17.9 | | 18.1 | | 18.3 | | 19.6 | | 19.9 | | | |  | | 2.6 | | 15.1 | | | | 0.11 (0.09; 0.14) | | <0.001 | |
|  | | AD ^b^ | | 83.9 | | 84.4 | | 85.0 | | 84.2 | | 84.9 | | 91.2 | | 91.9 | | | |  | | 8.0 | | 9.5 | | | | 0.33 (0.17; 0.50) | | <0.001 | |
|  | |  | |  | |  | |  | |  | |  | |  | |  | | | |  | |  | |  | | | |  | |  | |
| ICU ^C^ | | PD | | 22.8 | | 21.8 | | 22.0 | | 20.3 | | 20.6 | | 22.7 | | 23.3 | | | |  | | 0.5 | | 2.2 | | | | 0.02 (-0.06; 0.10) | | 0.337 | |
|  | | AD | | 99.2 | | 97.7 | | 93.3 | | 91.7 | | 97.2 | | 98.5 | | 95.3 | | | |  | | -3.9 | | -3.9 | | | | -0.05 (-0.30; 0.20) | | 0.684 | |
|  | |  | |  | |  | |  | |  | |  | |  | |  | | | |  | |  | |  | | | |  | |  | |
| General Ward | | PD | | 16.9 | | 17.1 | | 17.6 | | 17.9 | | 18.2 | | 19.4 | | 19.7 | | | |  | | 2.8 | | 16.6 | | | | 0.12 (0.10; 0.15) | | <0.001 | |
|  | | AD | | 83.9 | | 84.7 | | 85.7 | | 84.8 | | 85.2 | | 92.0 | | 93.0 | | | |  | | 9.1 | | 10.9 | | | | 0.37 (0.20; 0.54) | | <0.001 | |
|  | |  | |  | |  | |  | |  | |  | |  | |  | | | |  | |  | |  | | | |  | |  | |
| **Watch-antibiotics** | |  | |  | |  | |  | |  | |  | |  | |  | | | |  | |  | |  | | | |  | |  | |
| Whole hospital | | PD | | 36.2 | | 33.2 | | 30.0 | | 30.6 | | 29.3 | | 28.6 | | 28.5 | | | |  | | -7.6 | | -21.1 | | | | -0.29 (-0.36; -0.22) | | <0.001 | |
|  | | AD | | 175.5 | | 161.7 | | 142.5 | | 142.7 | | 135.6 | | 133.1 | | 131.7 | | | |  | | -43.8 | | -25.0 | | | | -1.74 (-2.12; -1.36) | | <0.001 | |
|  | |  | |  | |  | |  | |  | |  | |  | |  | | | |  | |  | |  | | | |  | |  | |
| ICU | | PD | | 73.1 | | 72.3 | | 70.4 | | 72.0 | | 70.9 | | 69.2 | | 69.1 | | | |  | | -4.0 | | -5.5 | | | | -0.16 (-0.27; -0.05) | | 0.005 | |
|  | | AD | | 318.6 | | 323.8 | | 299.6 | | 325.5 | | 334.9 | | 300.7 | | 283.2 | | | |  | | -35.4 | | -11.1 | | | | -1.03 (-2.01; -0.06) | | 0.039 | |
|  | |  | |  | |  | |  | |  | |  | |  | |  | | | |  | |  | |  | | | |  | |  | |
| General Ward | | PD | | 33.3 | | 30.3 | | 26.9 | | 27.1 | | 25.6 | | 25.3 | | 25.3 | | | |  | | -8.0 | | -23.9 | | | | -0.31 (-0.39; -0.24) | | <0.001 | |
|  | | AD | | 165.2 | | 150.2 | | 130.8 | | 128.0 | | 119.8 | | 119.6 | | 119.5 | | | |  | | -45.7 | | -27.7 | | | | -1.86 (-2.29; -1.44) | | <0.001 | |
|  | |  | |  | |  | |  | |  | |  | |  | |  | | | |  | |  | |  | | | |  | |  | |
|  | |  | |  | |  | |  | |  | |  | |  | |  | | | |  | |  | |  | | | |  | |  | |
| **Table S1 continued** |  | | **Pre-pandemic** | | | | | | **Pandemic** | | | | **Transition** | | | |  | | | | | | | |  | | | | | |  |
|  |  | | **2017** | | **2018** | | **2019** | | **2020** | | **2021** | | **2022** | | **2023** | | | |  | | **Difference**  **2017-23** | | **change (%)** | | | | Trend^a^ **(95%CI)** | | **Trend p-value** | |  |
| **Reserve-antibiotics** | | | | |  | |  | |  | |  | |  | |  | | | |  | |  | |  | | | |  | |  | |  |
| Whole hospital | PD | | 1.55 | | 1.63 | | 1.79 | | 2.04 | | 2.20 | | 2.16 | | 2.05 | | | |  | | 0.50 | | 32.3 | | | | 0.03 (0.02; 0.03) | | <0.001 | |  |
|  | AD | | 7.54 | | 7.92 | | 8.51 | | 9.50 | | 10.17 | | 10.03 | | 9.49 | | | |  | | 1.95 | | 25.9 | | | | 0.10 (0.07; 0.13) | | <0.001 | |  |
|  |  | |  | |  | |  | |  | |  | |  | |  | | | |  | |  | |  | | | |  | |  | |  |
| ICU | PD | | 8.10 | | 8.11 | | 8.64 | | 8.93 | | 9.39 | | 9.33 | | 8.42 | | | |  | | 0.32 | | 4.0 | | | | 0.04 (0.01; 0.07) | | 0.021 | |  |
|  | AD | | 35.33 | | 36.30 | | 36.74 | | 40.34 | | 44.35 | | 40.56 | | 34.53 | | | |  | | -0.80 | | -2.3 | | | | 0.12 (-0.08; 0.32) | | 0.224 | |  |
|  |  | |  | |  | |  | |  | |  | |  | |  | | | |  | |  | |  | | | |  | |  | |  |
| General Ward | PD | | 1.05 | | 1.14 | | 1.28 | | 1.46 | | 1.59 | | 1.58 | | 1.57 | | | |  | | 0.52 | | 49.5 | | | | 0.02 (0.02; 0.03) | | <0.001 | |  |
|  | AD | | 5.23 | | 5.66 | | 6.20 | | 6.88 | | 7.42 | | 7.48 | | 7.40 | | | |  | | 2.17 | | 41.5 | | | | 0.10 (0.08; 0.12) | | <0.001 | |  |
|  |  | |  | |  | |  | |  | |  | |  | |  | | | |  | |  | |  | | | |  | |  | |  |

^a^ Time trend can be interpreted as expected change in consumption between consecutive quarters. Values displayed represent point estimates, 95% CIs and p-values;  ^b^PD: DDD/100 patient days; AD: DDD/100 admissions; ^c^ICU, Intensive Care Unit
