# Supplementary material for: Kinetic Patterns of Antibiotic Consumption in German Acute Care Hospitals from 2017 to 2023
Source: Antibiotics (Basel). 2025 Mar 18;14(3):316. doi: 10.3390/antibiotics14030316 (PMC11939389; doi:10.3390/antibiotics14030316)
Supplement: Supplementary file 1 [file antibiotics-14-00316-s001.zip › Supplement Table S2.docx]

**Supplement Table S2. Antimicrobial consumption (DDD/100 patient days and DDD/100 admissions) of the AWaRe-categories from 2017 to 2023 subdivided into 3 phases: pre-pandemic phase (2017-2019), pandemic phase (2020-2021), transition phase (2022-2023): intra-phasic trends and inter-phasic changes of trend (pre-pandemic - pandemic and pandemic - transition).**

|  |  | **Pre-pandemic phase** | | |  |  | **Pandemic phase** | | | |  | **Transition phase** | | | |
| --- | --- | --- | --- | --- | --- | --- | --- | --- | --- | --- | --- | --- | --- | --- | --- |
|  |  | **2017-2019** | | |  |  | **2020-2021** | | | |  | **2022-2023** | | | |
| **Antibiotic group** |  | Diff^a^ 17-19 | change (%) | Trend | p-value |  | Diff 19-21 | Change (%) | Change of trend^b^ | p-value |  | Diff 21-23 | Change (%) | Change of trend^c^ | p-value |
| **Access-group** |  |  |  |  |  |  |  |  |  |  |  |  |  |  |  |
| Whole hospital | PD^d^ | 0.6 | 3.5 | 0.06 (-0.03; 0.15) | 0.192 |  | 0.4 | 2.5 | 0.04 (0.27; -0.19) | 0.895 |  | 1.6 | 8.5 | 0.003 (0.29; -0.28) | 1 |
|  | AD^d^ | 1.1 | 1.3 | 0.05 (-0.49; 0.59) | 0.585 |  | 0 | 0 | 0.28 (1.63; -1.07) | 0.86 |  | 6.9 | 8.1 | 0.07 (1.73; -1.59) | 0.993 |
|  |  |  |  |  |  |  |  |  |  |  |  |  |  |  |  |
| ICU^e^ | PD | -0.8 | -3.5 | -0.12 (-0.34; 0.11) | 0.288 |  | -1.4 | -6.2 | 0.29 (0.84; -0.27) | 0.401 |  | 2.7 | 13 | -0.06 (0.62; -0.74) | 0.973 |
|  | AD | -5.9 | -5.9 | -0.80 (-1.59; -0.01) | 0.047 |  | 3.8 | 4.1 | 2.40 (4.38; 0.42) | 0.016 |  | -1.8 | -1.9 | -2.33 (0.11; -4.77) | 0.063 |
|  |  |  |  |  |  |  |  |  |  |  |  |  |  |  |  |
| General Ward | PD | 0.7 | 4.3 | 0.07 (-0.01; 0.16) | 0.161 |  | 0.6 | 3.3 | 0.03 (0.25; -0.19) | 0.947 |  | 1.5 | 8.3 | 0.01 (0.28; -0.26) | 0.997 |
|  | AD | 1.8 | 2.1 | 0.13 (-0.42; 0.68) | 0.631 |  | -0.4 | -0.5 | 0.11 (1.50; -1.28) | 0.977 |  | 7.8 | 9.1 | 0.27 (1.98; -1.43) | 0.913 |
|  |  |  |  |  |  |  |  |  |  |  |  |  |  |  |  |
| **Watch-group** |  |  |  |  |  |  |  |  |  | |  |  |  |  |  |
| Whole hospital | PD | -6.2 | -17 | -0.78 (-0.90; -0.67) | <0.001 |  | -0.7 | -2.4 | 0.51 (0.78; 0.23) | <0.001 |  | -0.8 | -2.6 | 0.26 (0.60; -0.08) | 0.162 |
|  | AD | -33 | -18.8 | -4.21 (-4.89; -3.56) | <0.001 |  | -6.9 | -4.9 | 2.66 (4.28; 1.03) | <0.001 |  | -3.9 | -2.8 | 1.33 (3.33; -0.67) | 0.234 |
|  |  |  |  |  |  |  |  |  |  |  |  |  |  |  |  |
| ICU | PD | -2.6 | -3.6 | -0.38 (-0.78; 0.03) | 0.066 |  | 0.5 | 0.7 | 0.32 (1.33; -0.69) | 0.703 |  | -1.9 | -2.7 | -0.01 (1.23; -1.25) | 1 |
|  | AD | -19 | -6 | -2.57 (-4.89; -0.25) | 0.032 |  | 35.3 | 11.8 | 5.22 (11.02; -0.60) | 0.083 |  | -51.7 | -15.4 | -6.67 (0.48; -13.83) | 0.07 |
|  |  |  |  |  |  |  |  |  |  |  |  |  |  |  |  |
| General Ward | PD | -6.4 | -19.2 | -0.81 (-0.91; -0.71) | <0.001 |  | -1.3 | -4.9 | 0.50 (0.76; 0.25) | <0.001 |  | -0.3 | -1.1 | 0.33 (0.64; 0.02) | 0.038 |
|  | AD | -34.4 | -20.8 | -4.38 (-5.01; -3.74) | <0.001 |  | -10.9 | -8.4 | 2.56 (4.16; 0.96) | 0.002 |  | -0.3 | -0.3 | 1.91 (3.87; -0.06) | 0.059 |
|  |  |  |  |  |  |  |  |  |  |  |  |  |  |  |  |

| Table S2 continued |  | **Pre-pandemic phase** | | |  |  | **Pandemic phase** | | | |  | **Transition phase** | | | |
| --- | --- | --- | --- | --- | --- | --- | --- | --- | --- | --- | --- | --- | --- | --- | --- |
|  |  | **2017-2019** | | |  |  | **2020-2021** | | | |  | **2022-2023** | | | |
| **Antibiotic group** |  | Diff^a^ 17-19 | change (%) | Trend | p-value |  | Diff 19-21 | Change (%) | Change of trend^b^ | p-value |  | Diff 21-23 | Change (%) | Change of trend^c^ | p-value |
| **Reserve-group** |  |  |  |  |  |  |  |  |  |  |  |  |  |  |  |
| Whole hospital | PD | 0.24 | 15.5 | 0.032 (0.02; 0.05) | <0.001 |  | 0.41 | 22.9 | -0.00 (0.04; -0.04) | 1 |  | -0.15 | -6.8 | -0.06 (-0.01; -0.11) | 0.009 |
|  | AD | 0.97 | 12.9 | 0.13 (0.07; 0.19) | <0.001 |  | 1.66 | 19.5 | 0.00 (0.15; -0.15) | 0.998 |  | -0.68 | -6.7 | -0.27 (-0.09; -0.45) | 0.004 |
|  |  |  |  |  |  |  |  |  |  |  |  |  |  |  |  |
| ICU | PD | 0.54 | 6.7 | 0.09 (0.01; 0.16) | 0.021 |  | 0.75 | 8.7 | -0.01 (0.18; -0.19) | 0.995 |  | -0.97 | -10.3 | -0.10 (0.66; -0.86) | 0.003 |
|  | AD | 1.41 | 4 | 0.26 (-0.12; 0.64) | 0.644 |  | 7.61 | 20.7 | 0.47 (1.43; -0.45) | 0.439 |  | -9.82 | -22.1 | -2.33 (-1.16; -3.51) | <0.001 |
|  |  |  |  |  |  |  |  |  |  |  |  |  |  |  |  |
| General Ward | PD | 0.23 | 21.9 | 0.03 (0.02; 0.04) | <0.001 |  | 0.31 | 24.2 | 0.00 (0.03; -0.03) | 0.998 |  | -0.02 | -1.3 | -0.03 (0.001; -0.07) | 0.056 |
|  | AD | 0.97 | 18.5 | 0.12 (0.08; 0.17) | <0.001 |  | 1.22 | 19.7 | -0.00 (0.11; -0.12) | 0.997 |  | -0.02 | -0.3 | -0.14 (0.004; -0.28) | 0.058 |
|  |  |  |  |  |  |  |  |  |  |  |  |  |  |  |  |

^a^Diff: difference in DDD/100 PD and DDD/100 /AD; ^b^change of trend from the pre-pandemic to the pandemic phase; ^c^change of trend from the pandemic to the transition phase
^d^PD: DDD/100 patient days; AD: DDD/100 admissions; ^e^ICU, Intensive Care Unit
