# Supplementary material for: Kinetic Patterns of Antibiotic Consumption in German Acute Care Hospitals from 2017 to 2023
Source: Antibiotics (Basel). 2025 Mar 18;14(3):316. doi: 10.3390/antibiotics14030316 (PMC11939389; doi:10.3390/antibiotics14030316)
Supplement: Supplementary file 1 [file antibiotics-14-00316-s001.zip › Supplement Table S3.docx]

**Supplement Table S****3. Mean consumption levels (DDD/100 patient days and DDD/100 admissions) of the AWaRe-categories (WHO): differences between the pre-pandemic (2017-19) and the pandemic phase (2020-2021) and the pandemic and the transitional phase (2022-2023).**

|  |  | **Pre-pandemic phase** | **Pandemic phase** | **Transition phase** |  | **Difference** | |  | **Difference** | |
| --- | --- | --- | --- | --- | --- | --- | --- | --- | --- | --- |
|  |  | **2017-19** | **2020-21** | **2022-2023** |  | **pre-pandemic - pandemic** | |  | **pandemic - transition** | |
|  |  | Mean Value | Mean Value | Mean Value |  | Difference |  |  | Difference |  |
|  |  | (95%CI) | (95%CI) | (95%CI) |  | (95%CI) | p-value |  | (95%CI) | p-value |
| **Access-group** |  |  |  |  |  |  |  |  |  |  |
| Whole hospital | PD^a^ | 17.5 (17.2; 17.8) | 18.2 (17.8; 18.6) | 19.7 (19.4; 20.1) |  | 0.69 (1.18; 0.20) | 0.009 |  | 1.54 (2.08; 1.00) | < 0.001 |
|  | AD^a^ | 84.4 (82.6; 86.2) | 84.5(82.3; 86.7) | 91.5 (89.3; 93.7) |  | 0.08 (2.94; -2.78) | 0.952 |  | 7.01 (10.14; 3.87) | <0.001 |
|  |  |  |  |  |  |  |  |  |  |  |
| ICU^b^ | PD | 22.2 (21.4; 22.9) | 20.4 (19.5; 21.3) | 23.0 (22.0; 23.9) |  | -1.73 (-0.55; -2.91) | 0.006 |  | 2.52 (3.81; 1.24) | 0.001 |
|  | AD | 96.7 (94.0; 99.3) | 94.5 (91.2; 97.7) | 96.9 (93.7; 100.2) |  | -2.18 (2.02; -6.39) | 0.290 |  | 2.43 (7.03; -2.17) | 0.283 |
|  |  |  |  |  |  |  |  |  |  |  |
| General Ward | PD | 17.2 (16.9; 17.5) | 18.1 (17.7; 18.4) | 19.6 (19.2; 19.9) |  | 0.85(1.32; 0.39) | <0.001 |  | 1.52 (2.03; 1.01) | <0.001 |
|  | AD | 84.8 (82.9; 86.6) | 84.9 (82.7; 87.2) | 92.5 (90.2; 94.8) |  | 0.20 (3.14; -2.74) | 0.890 |  | 7.54 (10.76; 4.32) | <0.001 |
| **Watch-group** |  |  |  |  |  |  |  |  |  |  |
| Whole hospital | PD | 33.1 (32.7; 33.5) | 30 (29.5; 30.4) | 28.6 (28.1; 29.0) |  | -3.15 (-2.56; -3.73) | <0.001 |  | -1.4 (-0.75; -2.04) | <0.001 |
|  | AD | 159.8 (157.6; 162.) | 139.1 (136.5; 141.8) | 132.4 (129.7; 135.1) |  | -20.65 (-17.21; -24.09) | <0.001 |  | -6.72 (-2.95; -10.50) | 0.001 |
|  |  |  |  |  |  |  |  |  |  |  |
| ICU | PD | 71.9 (706; 73.3) | 71.5 (69.8; 73.1) | 69.1 (67.5; 70.8) |  | -0.47 (1.67; -2.61) | 0.652 |  | -2.34 (0.01; -4.68) | 0.050 |
|  | AD | 313.9 (306.1; 321.7) | 330.9 (321.3; 340.4) | 292.0 (282.4; 301.5) |  | 17.0 (29.33; 4.67) | 0.009 |  | -38.9 (-25.39; -52.41) | <0.001 |
|  |  |  |  |  |  |  |  |  |  |  |
| General Ward | PD | 30.1 (29.8; 30.5) | 26.3 (25.9; 26.7) | 25.3 (24.9; 25.7) |  | -3.81 (-3.28; -4.35) | <0,001 |  | -1.02 (-0.44; -1.61) | 0.002 |
|  | AD | 148.6 (146.5; 150.8) | 123.9 (121.2; 126.5) | 119.5 (116.9; 122.2) |  | -24.74 (-21.35; -28.15) | <0.001 |  | -4.32 (-0.60; -8.03) | 0.025 |
|  |  |  |  |  |  |  |  |  |  |  |

| Table S3 continued |  | **Pre-pandemic phase** | **Pandemic phase** | **Transition phase** |  | **Difference** | |  | **Difference** | |
| --- | --- | --- | --- | --- | --- | --- | --- | --- | --- | --- |
|  |  | **2017-19** | **2020-21** | **2022-2023** |  | **pre-pandemic - pandemic** | |  | **pandemic - transition** | |
|  |  | Mean Value | Mean Value | Mean Value |  | Difference |  |  | Difference |  |
|  |  | (95%CI) | (95%CI) | (95%CI) |  | (95%CI) | p-value |  | (95%CI) | p-value |
| **Reserve-group** |  |  |  |  |  |  |  |  |  |  |
| Whole hospital | PD | 1.66 (1.61; 1.71) | 2.12 (2.06; 2.18) | 2.11 (2.05; 2.17) |  | 0.46 (0.54; 0.38) | <0.001 |  | -0.02 (0.07; -0.10) | 0.694 |
|  | AD | 8.0 (7.80; 8.20) | 9.85 (9.61; 10.10) | 9.76 (9.52; 10.00) |  | 1.85 (2.16; 1.53) | <0.001 |  | -0.09 (0.25; -0.44) | 0.587 |
|  |  |  |  |  |  |  |  |  |  |  |
| ICU | PD | 8.29 (8.04; 8.54) | 9.16 (8.85; 9.47) | 8.88 (8.57; 9.18) |  | 0.87 (1.27; 0.47) | <0.001 |  | -0.28 (0.16; -0.72) | 0.193 |
|  | AD | 36.14 (34.86; 37.42) | 42.43 (40.86; 44.00) | 37.55 (35.98; 39.12) |  | 6.29 (8.31; 4.26) | <0.001 |  | -4.88 (-2.66; -7.10) | <0.001 |
|  |  |  |  |  |  |  |  |  |  |  |
| General Ward | PD | 1.16 (1.12; 1.20) | 1.52 (1.48; 1.57) | 1.58 (1.53; 1.62) |  | 0.36 (0.42; 0.31) | <0.001 |  | 0.05 (0.12; -0.01) | 0.097 |
|  | AD | 5.71 (5.55; 5.86) | 7.16 (6.97; 7.35) | 7.44 (7.25; 7.63) |  | 1.45 (1.70; 1.21) | <0.001 |  | 0.29 (0.55; 0.02) | 0.039 |
|  |  |  |  |  |  |  |  |  |  |  |

^a^PD: DDD/100 patient days; AD: DDD/100 admissions; ^b^ICU, Intensive Care Unit
