# Supplementary material for: Kinetic Patterns of Antibiotic Consumption in German Acute Care Hospitals from 2017 to 2023
Source: Antibiotics (Basel). 2025 Mar 18;14(3):316. doi: 10.3390/antibiotics14030316 (PMC11939389; doi:10.3390/antibiotics14030316)
Supplement: Supplementary file 1 [file antibiotics-14-00316-s001.zip › Supplement Table S4.docx]

**Supplement Table S4.** **Trends of antimicrobial consumption (DDD/100 patient days) of selected antibiotic classes/substances from 2017 to 2023.**

|  | **Pre-pandemic** | | | **Pandemic** | | **Transition** | |  |  |  |  |  |
| --- | --- | --- | --- | --- | --- | --- | --- | --- | --- | --- | --- | --- |
|  | **2017** | **2018** | **2019** | **2020** | **2021** | **2022** | **2023** |  | **Difference** | **change** | **Trend** ^a^ | **Trend** |
|  |  |  |  |  |  |  |  |  | **17-23** | **(%)** |  | **p-value** |
| J01C-Penicillins | 15.36 | 16.58 | 18.23 | 19.19 | 19.88 | 21.72 | 22.36 |  | 7.00 | 45.6 | 0.294 (0.267; 0.320) | <0.001 |
| J01CA-Penicillins with extended spectrum | 1.77 | 1.70 | 1.78 | 1.95 | 1.96 | 2.24 | 2.12 |  | 0.35 | 19.8 | 0.021 (0.014; 0.026) | <0.001 |
| J01CE-Beta-lactamase sensitive penicillins | 1.48 | 1.54 | 1.65 | 1.51 | 1.52 | 1.71 | 1.91 |  | 0.43 | 29.1 | 0.013 (0.006; 0.020) | <0.001 |
| J01CF-Beta-lactamase resistant penicillins | 1.38 | 1.57 | 1.71 | 1.80 | 2.17 | 2.25 | 2.05 |  | 0.67 | 48.6 | 0.034 (0.026; 0.043) | <0.001 |
| J01CR01/02/04-Aminopenicillins/BLI^b^ | 6.60 | 7.07 | 7.72 | 7.88 | 7.87 | 8.86 | 9.29 |  | 2.69 | 40.8 | 0.106 (0.084; 0.128) | <0.001 |
| J01CR05-Piperacillin/tazobactam | 4.13 | 4.70 | 5.36 | 6.06 | 6.36 | 6.66 | 6.99 |  | 2.86 | 69.2 | 0.120 (0.110; 0.131) | <0.001 |
|  |  |  |  |  |  |  |  |  |  |  |  |  |
| J01DB/DC/DD/DE-Cephalosporins | 16.17 | 13.67 | 12.26 | 12.19 | 11.54 | 10.91 | 10.67 |  | -5.50 | -34.0 | -0.203 (-0.245; -0.161) | 0.003 |
| J01DB-First-generation cephalosporins | 1.09 | 1.15 | 1.33 | 1.50 | 1.52 | 1.59 | 1.74 |  | 0.65 | 59.6 | 0.027 (0.023; 0.030) | <0.001 |
| J01DC-Second-generation cephalosporins | 10.19 | 7.70 | **5.77** | 5.07 | 4.35 | 3.84 | 3.57 |  | -6.62 | -65.0 | -0.258 (-0.304; -0.213) | <0.001 |
| J01DD-Third-generation cephalosporins | 4.80 | 4.73 | 5.07 | 5.55 | 5.61 | 5.42 | 5.29 |  | 0.49 | 10.2 | 0.030 (0.016; 0.044) | <0.001 |
| J01DE-Fourth-generation cephalosporins | 0.09 | 0.09 | 0.09 | 0.08 | 0.07 | 0.07 | 0.08 |  | -0.01 | -10.8 | -0.001(-0.002; -0.001) | <0.001 |
|  |  |  |  |  |  |  |  |  |  |  |  |  |
| J01DH-Carbapenems | 2.89 | 2.92 | 3.08 | 3.53 | 3.64 | 3.53 | 3.50 |  | 0.61 | 21.1 | 0.032 (0.023; 0.0422) | <0.001 |
|  |  |  |  |  |  |  |  |  |  |  |  |  |
| J01FA-Macrolides | 4.28 | 3.65 | 3.38 | 3.65 | 2.81 | 2.85 | 2.96 |  | -1.32 | -30.8 | -0.054 (-0.077; -0.030) | <0.001 |
| J01FA01-Erythromycin | 0.32 | 0.24 | 0.19 | 0.27 | 0.27 | 0.27 | 0.26 |  | -0.06 | -18.8 | 0.000 (-0.002; 0.002) | 0.876 |
| J01FA09-Clarithromycin | 3.46 | 2.81 | 2.41 | 2.45 | 1.70 | 1.43 | 1.05 |  | -2.41 | -69.7 | -0.095(-0.112; -0.0772) | <0.001 |
| J01FA10-Azithromycin | 0.23 | 0.40 | 0.59 | 0.78 | 0.71 | 1.04 | 1.56 |  | 1.33 | 578.3 | 0.048 (0.039; 0.057) | <0.001 |
|  |  |  |  |  |  |  |  |  |  |  |  |  |
| J01MA-Fluoroquinolones | 6.89 | 6.44 | 4.24 | 3.43 | 3.16 | 3.06 | 2.88 |  | -4.01 | -58.2 | -0.177 (-0.214; -0.140) | <0.001 |
| J01MA02-Ciprofloxcin | 4.45 | 4.00 | 2.48 | 1.89 | 1.73 | 1.69 | 1.53 |  | -2.92 | -65.6 | -0.126 (-0.152; -0.100) | <0.001 |
| J01MA12-Levofloxacin | 1.61 | 1.77 | 1.23 | 1.06 | 1.03 | 0.99 | 0.99 |  | -0.62 | -38.5 | -0.032 (-0.041; -0.023) | <0.001 |
| J01MA14-Moxifloxacin | 0.83 | 0.68 | 0.52 | 0.48 | 0.39 | 0.38 | 0.36 |  | -0.47 | -56.6 | -0.019 (-0.023; -0.015) | <0.001 |
| **Table S4 continued** | **Pre-pandemic** | | | **Pandemic** | | **Transition** | |  |  |  |  |  |
|  | **2017** | **2018** | **2019** | **2020** | **2021** | **2022** | **2023** |  | **Difference** | **change** | **Trend** | **Trend** |
|  |  |  |  |  |  |  |  |  | **17-23** | **(%)** |  | **p-value** |
| J01XA-Glycopeptides | 1.15 | 1.15 | 1.08 | 1.22 | 1.21 | 1.16 | 1.15 |  | 0.00 | 0.00 | 0.001 (-0.002; 0.004) | 0.356 |
| J01XB-Polymyxins (parenteral) | 0.04 | 0.03 | 0.03 | 0.02 | 0.02 | 0.02 | 0.02 |  | -0.02 | -57.1 | -0.001 (-0.001; -0.0006) | <0.001 |
| J01XX01-Fosfomycin (parenteral) | 0.38 | 0.47 | 0.54 | 0.62 | 0.67 | 0.65 | 0.62 |  | 0.24 | 63.2 | 0.011 (0.008; 0.014) | <0.001 |
| J01XX08-Linezolid | 0.69 | 0.70 | 0.74 | 0.87 | 0.89 | 0.85 | 0.85 |  | 0.16 | 23.2 | 0.008(0.005; 0.0113) | <0.001 |
| J01XX09-Daptomycin | 0.25 | 0.26 | 0.30 | 0.34 | 0.39 | 0.41 | 0.38 |  | 0.13 | 52.0 | 0.007 (0.005; 0.009) | <0.001 |
| J01AA12-Tigecyclin | 0.14 | 0.13 | 0.12 | 0.12 | 0.14 | 0.13 | 0.12 |  | -0.02 | -17.5 | -0.001 (-0.001; 0.0001) | 0.112 |
| J01DD52-Ceftazidim/avibactam | 0.01 | 0.01 | 0.02 | 0.02 | 0.04 | 0.03 | 0.03 |  | 0.02 | 355.1 | 0.001 (0.001; 0.0014) | <0.001 |
| J01DF-Monobactams | 0.000 | 0.002 | 0.001 | 0.001 | 0.003 | 0.008 | 0.008 |  | 0.01 | 2672.5 | 0.0003(0.0002; 0.0004) | <0.001 |
| J01DI04-Cefiderocol | 0 | 0 | 0 | 0.000 | 0.006 | 0.010 | 0.008 |  | 0.01 | n.a.^c^ | n.a. | n.a. |
| J01DI54-Ceftolozan/avibactam | 0.006 | 0.011 | 0.010 | 0.014 | -0.001 | 0.011 | 0.012 |  | 0.01 | 122 | 0.0001(-0.0002; 0.0003) | 0.582 |

^a^ Time trend can be interpreted as expected change in consumption between consecutive quarters. Values displayed represent point estimates, 95% CIs and p-values; ^b^BLI, betalactamase-inhibitor; ^c^n.a., not applicable
