# Supplementary material for: Kinetic Patterns of Antibiotic Consumption in German Acute Care Hospitals from 2017 to 2023
Source: Antibiotics (Basel). 2025 Mar 18;14(3):316. doi: 10.3390/antibiotics14030316 (PMC11939389; doi:10.3390/antibiotics14030316)
Supplement: Supplementary file 1 [file antibiotics-14-00316-s001.zip › Supplement Table S5.docx]

**Supplement Table S5. Antimicrobial consumption (DDD/100 patient days) of selected antibiotics from 2017 to 2023 subdivided into pre-pandemic phase (2017-2019), pandemic phase (2020-2021), transition phase (2022-2023): display of intra-phasic trends and inter-phasic changes of trend (pre-pandemic-pandemic and pandemic-transition)**

|  | **Pre-pandemic phase** | | | | | | | |  | **Pandemic phase** | | | | | | | | |  | **Transition phase** | | | | | | | | |  |
| --- | --- | --- | --- | --- | --- | --- | --- | --- | --- | --- | --- | --- | --- | --- | --- | --- | --- | --- | --- | --- | --- | --- | --- | --- | --- | --- | --- | --- | --- |
|  | **2017-2019** | | | | | | | |  | **2020-2021** | | | | | | | | |  | **2022-2023** | | | | | | | | |  |
| **Antibiotic** | **Diff.^a^ 17-19** | | | **change (%)** | **Trend** | | **p-value** | |  | **Diff. 19-21** | | | **change (%)** | | **Change of trend^b^** | | **p-value** | |  | **Diff. 21-23** | | | **change (%)** | | **Change of trend^c^** | | **p-value** | |  |
| **J01C-Penicillins** | | | | | | | | |  |  | | |  | |  | |  | |  |  | | |  | |  | |  | |  |
|  | 2.87 | | | 18.7 | 0.330  (0.272; 0.433) | | <0.001 | |  | 1.65 | | | 9.1 | | 0.108  (0.149; -0.344) | | 0.550 | |  | *2.48* | | | 12.5 | | 0.008  (0.325; -0.309) | | 1.000 | |  |
| **J01CA-Penicillins with extended spectrum** | | | | | | |  | |  |  | | |  | |  | |  | |  |  | | |  | |  | |  | |  |
|  | 0.01 | | | 0.6 | -0.003 (-0.024;0.018) | | 0.754 | |  | 0.18 | | | 10.1 | | 0.010  (0.062; -0.043) | | 0.887 | |  | 0.16 | | | 8.2 | | -0.019  (0.046; -0.083) | | 0.749 | |  |
| **J01CE-Beta-lactamase sensitive penicillins** | | | | | | | | |  |  | | |  | |  | |  | |  |  | | |  | |  | |  | |  |
|  | 0.17 | | | 11.5 | 0.025  (0.006; 0.043) | | 0.012 | |  | -0.13 | | | -7.9 | | -0.025 (0.022; -0.071) | | 0.389 | |  | 0.39 | | | 25.7 | | 0.035  (0.093; -0.022) | | 0.284 | |  |
| **J01CF-Beta-lactamase resistant penicillins** | | | | | | | | |  |  | | |  | |  | |  | |  |  | | |  | |  | |  | |  |
|  | 0.33 | | | 23.9 | 0.043  (0.026; 0.060) | | <0.001 | |  | 0.46 | | | 26.9 | | 0.048  (0.091; 0.006) | | 0.025 | |  | -0.12 | | | -5.5 | | -0.149  (-0.097; -0.202) | | <0.001 | |  |
| **J01CR01/02/04-Aminopenicillins/beta-lactamase inhibitor** | | | | | | |  | |  |  | | |  | |  | |  | |  |  | | |  | |  | |  | |  |
|  | 1.12 | | | 17 | 0.118  (0.038; 0.197) | | 0.006 | |  | 0.15 | | | 1.9 | | -0.079  (0.120; -0.279) | | 0.582 | |  | 1.42 | | | 18 | | 0.128  (0.373; -0.118) | | 0.402 | |  |
| **J01CR05-Piperacillin/tazobactam** | | | | |  | |  | |  |  | | |  | |  | |  | |  |  | | |  | |  | |  | |  |
|  | 1.23 | | | 29.8 | 0.148  (0.124; 0.172) | | <0.001 | |  | 1.00 | | | 18.7 | | -0.062 (-0.003; -0.122) | | 0.040 | |  | 0.63 | | | 9.9 | | 0.013  (0.087; -0.061) | | 0.896 | |  |
| **J01DB/C/D/E-Cephalosporins** | | | | | | | | |  |  | | |  | |  | |  | |  |  | | |  | |  | |  | |  |
|  | -3.91 | | | -24.2 | -0.481  (-0.565; -0.398) | | <0.001 | |  | -0.72 | | | -5.9 | | 0.320  (0.530; 0.111) | | 0.003 | |  | -0.87 | | | -7.5 | | 0.069  (0.327; -0.189) | | 0.778 | |  |
| **J01DB-First-generation cephalosporins** | | | | | | | | |  |  | | |  | |  | |  | |  |  | | |  | |  | |  | |  |
|  | 0.24 | | | 22 | 0.030  (0.020; 0.040) | | <0.001 | |  | 0.19 | | | 14.3 | | -0.033  (-0.008; -0.057) | | 0.008 | |  | 0.22 | | | 14.5 | | 0.042  (0.072; 0.012) | | 0.006 | |  |
|  |  |  |  | | |  | |  | | |  |  | |  | |  | |  | | |  |  | |  | |  | |  | |
| **Table S5** | **Pre-pandemic phase** | | | | | | | |  | **Pandemic phase** | | | | | | | | |  | **Transition phase** | | | | | | | | |  |
| **continued** | **2017-2019** | | | | | | | |  | **2020-2021** | | | | | | | | |  | **2022-2023** | | | | | | | | |  |
| **Antibiotic** | **Diff.^a^ 17-19** | | | **change (%)** | **Trend** | | **p-value** | |  | **Diff. 19-21** | | | **change (%)** | | **Change of trend^b^** | | **p-value** | |  | **Diff. 21-23** | | | **change (%)** | | **Change of trend^c^** | | **p-value** | |  |
| **J01DC-Second-generation cephalosporins** | | | | | | | | |  |  | | |  | |  | |  | |  |  | | |  | |  | |  | |  |
|  | -4.42 | | | -43.4 | -0.544  (-0.607; -0.481) | | <0.001 | |  | -1.42 | | | -24.6 | | 0.364  (0.521; 0.207) | | <0.001 | |  | -0.78 | | | -17.9 | | 0.081  (0.274; -0.113) | | 0.551 | |  |
| **J01DD-Third-generation cephalosporins** | | | | | | | | |  |  | | |  | |  | |  | |  |  | | |  | |  | |  | |  |
|  | 0.27 | | | 5.6 | 0.033  (-0.002; 0.067) | | 0.06 | |  | 0.54 | | | 10.7 | | -0.001  (0.076; -0.095) | | 0.959 | |  | -0.32 | | | -5.7 | | -0.055  (0.050; -0.160) | | 0.397 | |  |
| **J01DE-Fourth-generation cephalosporins** | | | | | | | | |  |  | | |  | |  | |  | |  |  | | |  | |  | |  | |  |
|  | 0 | | | 1.5 | 0.0002  (-0.002; 0.002) | | 0.797 | |  | -0.02 | | | -18.8 | | -0.002  (0.003; -0.006) | | 0.571 | |  | 0.01 | | | 8.2 | | 0.002  (0.007; -0.004) | | 0.775 | |  |
| **J01DH-Carbapenems** | | | |  |  | |  | |  |  | | |  | |  | |  | |  |  | | |  | |  | |  | |  |
|  | 0.19 | | | 6.6 | 0.023  (0.001; 0.045) | | 0.043 | |  | 0.56 | | | 18.2 | | 0.006  (0.061; -0.050) | | 0.966 | |  | -0.14 | | | -3.8 | | -0.031  (0.037; -0.010) | | 0.491 | |  |
| **J01FA-Macrolides** | | | | | | | | |  |  | | |  | |  | |  | |  |  | | |  | |  | |  | |  |
|  | -0.9 | | | -21 | -0.130  (-0.206; -0.055) | | 0,002 | |  | -0.57 | | | -16.9 | | -0.038  (0.151; -0.226) | | 0.868 | |  | 0.15 | | | 5.3 | | 0.236  (0.467; 0.004) | | 0.046 | |  |
| **J01FA01-Erythromycin** | | | | | | | | |  |  | | |  | |  | |  | |  |  | | |  | |  | |  | |  |
|  | -0.13 | | | -40.6 | -0.015  (-0.020; -0.010) | | <0.001 | |  | 0.08 | | | 42.1 | | 0.018  (0.030; 0.005) | | 0.005 | |  | -0.01 | | | -3.7 | | -0.007  (0.009; -0.022) | | 0.508 | |  |
| **J01FA09-Clarithromycin** | | | | | | | | |  |  | | |  | |  | |  | |  |  | | |  | |  | |  | |  |
|  | -1.05 | | | -30.3 | -0.145  (-0.207; -0.082) | | <0.001 | |  | -0.71 | | | -29.5 | | -0.012  (0.145; -0.169) | | 0.98 | |  | -0.65 | | | -38.2 | | 0.096  (0.290; -0.097) | | 0.432 | |  |
| **J01FA10-Azithromycin** | | | | | | | | |  |  | | |  | |  | |  | |  |  | | |  | |  | |  | |  |
|  | 0.36 | | | 156.5 | 0.042  (0.022; 0.061) | | <0.001 | |  | 0.12 | | | 20.3 | | -0.052  (-0.003; -0.102) | | 0.038 | |  | 0.85 | | | 119.7 | | 0.146  (0.208; 0.085) | | <0.001 | |  |
|  |  |  |  | | |  | |  | | |  |  | |  | |  | |  | | |  |  | |  | |  | |  | |
| **Table S5** | **Pre-pandemic phase** | | | | | | | |  | **Pandemic phase** | | | | | | | | |  | **Transition phase** | | | | | | | | |  |
| **continued** | **2017-2019** | | | | | | | |  | **2020-2021** | | | | | | | | |  | **2022-2023** | | | | | | | | |  |
| **Antibiotic** | **Diff.^a^ 17-19** | | | **change (%)** | **Trend** | | **p-value** | |  | **Diff. 19-21** | | | **change (%)** | | **Change of trend^b^** | | **p-value** | |  | **Diff. 21-23** | | | **change (%)** | | **Change of trend^c^** | | **p-value** | |  |
| **J01MA-Fluoroquinolones** | | | | | | | | |  |  | | |  | |  | |  | |  |  | | |  | |  | |  | |  |
|  | -2.65 | | | -38.5 | -0.330  (-0.408; -0.252) | | <0.001 | |  | -1.08 | | | -25.5 | | 0.261  (0.457; 0.066) | | 0.008 | |  | -0.28 | | | -8.9 | | 0.011  (0.252; -0.230) | | 0.992 | |  |
| **J01MA02-Ciprofloxacin** | | | | | | | | |  |  | | |  | |  | |  | |  |  | | |  | |  | |  | |  |
|  | -1.97 | | | -44.3 | -0.240  (-0.291; -0.189) | | <0.001 | |  | -0.75 | | | -30.2 | | 0.189  (0.318; 0.060) | | 0.004 | |  | -0.2 | | | -11.6 | | -0.006  (0.153; -0.164) | | 0.995 | |  |
| **J01MA12-Levofloxacin** | | | | | | | | |  |  | | |  | |  | |  | |  |  | | |  | |  | |  | |  |
|  | -0.38 | | | -23.6 | -0.050  (-0.079; -0.022) | | <0.001 | |  | -0.2 | | | -16.3 | | 0.052  (0.123; -0.019) | | 0.181 | |  | -0.04 | | | -3.9 | | 0.001  (0.089; -0.087) | | 1.000 | |  |
| **J01MA14-Moxifloxacin** | | | | | | | | |  |  | | |  | |  | |  | |  |  | | |  | |  | |  | |  |
|  | -0.31 | | | -37.3 | -0.039  (-0.048; -0.030) | | <0.001 | |  | -0.13 | | | -25 | | 0.020  (0.043; -0.002) | | 0.081 | |  | -0.03 | | | -7.7 | | 0.016  (0.044; -0.012) | | 0.320 | |  |
| **J01XA-Glycopeptides** | | | | | | | | |  |  | | |  | |  | |  | |  |  | | |  | |  | |  | |  |
|  | -0.07 | | | -6.1 | -0.009  (-0.017; -0.002) | | 0.018 | |  | 0.13 | | | 12 | | 0.004  (0.023; -0.015) | | 0.865 | |  | -0.06 | | | -5 | | 0.005  (0.029; -0.018) | | 0.831 | |  |
| **J01XB-Polymyxines (parenteral)** | | | | | | | | |  |  | | |  | |  | |  | |  |  | | |  | |  | |  | |  |
|  | -0.01 | | | -34.1 | -0.002  (-0.002; -0.001) | | 0.001 | |  | 0 | | | -7.7 | | 0.001  (0.003; -0.001) | | 0.674 | |  | -0.01 | | | -29.5 | | -0.0003  (0.002; -0.003) | | 0.966 | |  |
| **J01XX01-Fosfomycin (parenteral)** | | | | | | | | |  |  | | |  | |  | |  | |  |  | | |  | |  | |  | |  |
|  | 0.16 | | | 42.1 | 0.021  (0.017; 0.025) | | <0.001 | |  | 0.13 | | | 24.1 | | -0.010  (0.001; -0.020) | | 0.082 | |  | -0.05 | | | -7.5 | | -0.021  (-0.008; -0.034) | | 0.002 | |  |
| **J01XX08-Linezolid** | | | | | | | | |  |  | | |  | |  | |  | |  |  | | |  | |  | |  | |  |
|  | 0.05 | | | 7.2 | 0.007  (-0.001; 0.014) | | 0.095 | |  | 0.15 | | | 20.3 | | -0.003  (0.016; -0.023) | | 0.902 | |  | -0.04 | | | -4.5 | | -0.004  (0.020; -0.028) | | 0.896 | |  |
| **J01XX09-Daptomycin** | | | | | | | | |  |  | | |  | |  | |  | |  |  | | |  | |  | |  | |  |
|  | 0.05 | | | 20 | 0.006  (-0.0003; 0.013) | | 0.062 | |  | 0.09 | | | 30 | | 0.006  (0.022; -0.011) | | 0.663 | |  | -0.01 | | | -2.6 | | -0.023  (-0.003; -0.043) | | 0.022 | |  |
|  |  |  |  | | |  | |  | | |  |  | |  | |  | |  | | |  |  | |  | |  | |  | |
| **Table S5** | **Pre-pandemic phase** | | | | | | | |  | **Pandemic phase** | | | | | | | | |  | **Transition phase** | | | | | | | | |  |
| **continued** | **2017-2019** | | | | | | | |  | **2020-2021** | | | | | | | | |  | **2022-2023** | | | | | | | | |  |
| **Antibiotic** | **Diff.^a^ 17-19** | | | **change (%)** | **Trend** | | **p-value** | |  | **Diff. 19-21** | | | **change (%)** | | **Change of trend^b^** | | **p-value** | |  | **Diff. 21-23** | | | **change (%)** | | **Change of trend^c^** | | **p-value** | |  |
| **J01AA12-Tigecyclin** | | | | | | | | |  |  | | |  | |  | |  | |  |  | | |  | |  | |  | |  |
|  | -0.03 | | | -18.4 | -0.003  (-0.005; -0.001) | | 0.009 | |  | 0.02 | | | 20.3 | | 0.007  (0.012; 0.001) | | 0.019 | |  | -0.02 | | | -15.9 | | -0.007  (-0.001; -0.014) | | 0.033 | |  |
| **J01DD52-Ceftazidim/avibactam** | | | | | | | | |  |  | | |  | |  | |  | |  |  | | |  | |  | |  | |  |
|  | 0.01 | | | 203.7 | 0.002  (0.001; 0.002) | | 0.001 | |  | 0.02 | | | 98.5 | | 0.001  (0.003; -0.001) | | 0.605 | |  | -0.01 | | | -24.5 | | -0.004  (-0.001; -0.006) | | 0.004 | |  |
| **J01DF-Monobactams** | | | | | | | | |  |  | | |  | |  | |  | |  |  | | |  | |  | |  | |  |
|  | 0 | | | 169.5 | 0.0001  (-0.0002; 0.0004) | | 0.71 | |  | 0 | | | 228.6 | | 0.0004  (0.001; -0.0003) | | 0.345 | |  | 0.01 | | | 213 | | -0.0002  (0.001; -0.001) | | 0.784 | |  |
| **J01DI04-Cefiderocol** | | | | | | | | |  |  | | |  | |  | |  | |  |  | | |  | |  | |  | |  |
|  | n.a.**^d^** | | | n.a. | n.a. | | n.a. | |  | n.a. | | | n.a. | | 0.001  (0.001; 0.002) | | <0.001 | |  | 0 | | | 27.1 | | -0.002  (-0.001; -0.003) | | <0.001 | |  |
| **J01DI54-Ceftolozan/tazobactam** | | | | | | | | |  |  | | |  | |  | |  | |  |  | | |  | |  | |  | |  |
|  | 0 | | | 81.1 | 0.0004  (-0.0004; 0.001) | | 0.304 | |  | n.a. | | | n.a. | | n.a. | | n.a. | |  | n.a. | | | n.a. | | n.a. | | n.a. | |  |

^a^Diff: difference in DDD/100 patient days; ^b^change of trend from the pre-pandemic to the pandemic phase; ^c^change of trend from the pandemic to the transition phase;
^d^ n.a., not applicable;
