# Supplementary material for: Kinetic Patterns of Antibiotic Consumption in German Acute Care Hospitals from 2017 to 2023
Source: Antibiotics (Basel). 2025 Mar 18;14(3):316. doi: 10.3390/antibiotics14030316 (PMC11939389; doi:10.3390/antibiotics14030316)
Supplement: Supplementary file 1 [file antibiotics-14-00316-s001.zip › Supplement Table S6.docx]

**Supplement Table S6. Mean consumption levels (DDD/100 patient days) of selected antibiotics classes/substances: difference between the pre-pandemic (2017-19) and the pandemic phase (2020-2021) and between the pandemic and the transitional phase (2022-2023).**

|  | **Pre-pandemic phase** | **Pandemic phase** | **Transition phase** | **Difference** | | **Difference** | |
| --- | --- | --- | --- | --- | --- | --- | --- |
|  | **2017-19** | **2020-21** | **2022-2023** | **pre-pandemic- pandemic** | | **pandemic-transition** | |
|  | **Mean Value (95%CI)** | **Mean Value (95%CI)** | **Mean Value (95%CI)** | **Difference (95%CI)** | **p-value** | **Difference (95%CI)** | **p-value** |
| **J01C-Penicillins** | | | |  |  |  |  |
|  | 16.72 (16.37; 17.06) | 19.52 (19.09; 19.94) | 22.04 (21.61; 22.45) | 2.80 (3.34; 2.25) | <0.001 | 2.52 (1.91; 3.12) | <0.001 |
| **J01CA-penicillins with extended spectrum** | | | |  |  |  |  |
|  | 1.75 (1.68; 1.82) | 1.95 (1.87; 2.04) | 2.18 (2.09; 2.26) | 0.20 (0.32; 0.09) | 0.001 | 0.22 (0.34; 0.10) | 0.001 |
| **J01CE-Beta-lactamase sensitive penicillins** | | | |  |  |  |  |
|  | 1.56 (1.50; 1.62) | 1.51 (1.43; 1.59) | 1.81 (1.73; 1.89) | -0.05 (0.05; -0.15) | 0.319 | 0.3 (0.41; 0.19) | <0.001 |
| **J01CF-Beta-lactamase resistant penicillins** | | | |  |  |  |  |
|  | 1.56 (1.50; 1.61) | 1.99 (1.92; 2.06) | 2.15 (2.08; 2.22) | 0.43 (0.52; 0.34) | <0.001 | 0.16 (0.26; 0.06) | 0.003 |
| **J01CR01/02/04-Aminopenicillins/beta-lactamase inhibitors** | | | |  |  |  |  |
|  | 7.12 (6.85; 7.39) | 7.85 (7.53; 8.18) | 9.07 (8.75; 9.40) | 0.73 (1.16; 0.31) | 0.002 | 1.22 (1.68; 0.76) | <0.001 |
| **J01CR05-Piperacillin/tazobactam** | | | |  |  |  |  |
|  | 4.74 (4.66; 4.82) | 6.21 (6.11; 6.31) | 6.82 (6.72; 6.92) | 1.48 (1.60; 1.35) | <0.001 | 0.61 (0.75; 0.47) | <0.001 |
| **J01DB/DC/DD/DE-Cephalosporins** | | | |  |  |  |  |
|  | 14.03 (13.75; 14.31) | 11.87 (11.53; 12.22) | 10.79 (10.45; 11.14) | -2.16 ( -1.71; -2.60) | <0.001 | -1.08 ( -0.59; -1.56) | <0.001 |
| **J01DB-First-generation cephalosporins** | | | |  |  |  |  |
|  | 1.19 (1.16; 1.22) | 1.51 (1.47; 1.55) | 1.66 (1.62; 1.70) | 0.32 (0.37; 0.27) | <0.001 | 0.15 (0.21; 0.10) | <0.001 |
| **J01DC-Second-generation cephalosporins** | | | |  |  |  |  |
|  | 7.88 (7.67; 8.09) | 4.70 (4.45; 4.96) | 3.71 (3.45; 3.97) | -3.18 ( -2.84; -3.51) | <0.001 | -1.00 ( -0.63; -1.36) | <0.001 |
| **J01DD-Third-generation cephalosporins** | | | |  |  |  |  |
|  | 4.87 (4.75; 4.98) | 5.58 (5.44; 5.72) | 5.35 (5.21; 5.49) | 0.71 (0.89; 0.53) | <0.001 | -0.23 ( -0.03; -0.42) | 0.027 |
| **J01DE-Fourth-generation cephalosporins** | | | |  |  |  |  |
|  | 0.09 (0.08; 0.10) | 0.08 (0.07; 0.08) | 0.07 (0.06; 0.08) | -0.01 ( -0.00; -0.02) | 0.006 | -0.01 (0.00; -0.02) | 0.143 |
|  |  |  |  |  |  |  |  |
| Table S6 | **Pre-pandemic phase** | **Pandemic phase** | **Transition phase** | **Difference** | | **Difference** | |
| continued | **2017-19** | **2020-21** | **2022-2023** | **pre-pandemic- pandemic** | | **pandemic-transition** | |
|  | **Mean Value (95%CI)** | **Mean Value (95%CI)** | **Mean Value (95%CI)** | **Difference (95%CI)** | **p-value** | **Difference (95%CI)** | **p-value** |
| **J01DH-Carbapenems** | |  |  |  |  |  |  |
|  | 2.96 (2.89; 3.04) | 3.59 (3.5; 3.68) | 3.51 (3.42; 3.60) | 0.63 (0.75; 0.51) | <0.001 | -0.08 (0.05; -0.21) | 0.218 |
| **J01FA-Macrolides** | | | |  |  |  |  |
|  | 3.75 (3.50; 4.00) | 3.22 (2.91; 3.53) | 2.90 (2.59; 3.21) | -0.53 ( -0.13; -0.93) | 0.012 | -0.32 (0.12; -0.76) | 0.14 |
| **J01FA01-Erythromycin** | | | |  |  |  |  |
|  | 0.25 (0.23; 0.27) | 0.27 (0.25; 0.29) | 0.27 (0.25; 0.29) | 0.02 (0.05; -0.01) | 0.138 | 0.00 (0.03; -0.03) | 0.885 |
| **J01FA09-Clarithromycin** | | | |  |  |  |  |
|  | 2.88 (2.67; 3.09) | 2.07 (1.81; 2.33) | 1.24 (0.98; 1.50) | -0.81 ( -0.48; -1.14) | <0.001 | -0.83 ( -0.46; -1.19) | <0.001 |
| **J01FA10-Azithromycin** | | | |  |  |  |  |
|  | 0.41 (0.34; 0.48) | 0.74 (0.66; 0.82) | 1.30 (1.22; 1.38) | 0.33 (0.44; 0.23) | <0.001 | 0.56 (0.68; 0.44) | <0.001 |
| **J01MA-Fluoroquinolones** | | | |  |  |  |  |
|  | 5.85 (5.59; 6.11) | 3.29 (2.97; 3.61) | 2.97 (2.65; 3.29) | -2.56 ( -2.15; -2.98) | <0.001 | -0.32 (0.14; -0.77) | 0.16 |
| **J01MA02-Ciprofloxacin** | | | |  |  |  |  |
|  | 3.64 (3.47; 3.81) | 1.81 (1.60; 2.02) | 1.61 (1.40; 1.82) | -1.83 ( -1.56; -2.10) | <0.001 | -0.2 (0.10; -0.50) | 0.174 |
| **J01MA12-Levofloxacin** | | | |  |  |  |  |
|  | 1.53 (1.44; 1.63) | 1.04 (0.93; 1.16) | 0.99 (0.88; 1.11) | -0.49 ( -0.34; -0.64) | <0.001 | -0.05 (0.12; -0.22) | 0.533 |
| **J01MA14-Moxifloxacin** | | | |  |  |  |  |
|  | 0.67 (0.64; 0.70) | 0.44 (0.40; 0.47) | 0.37 (0.33; 0.41) | -0.24 ( -0.19; -0.29) | <0.001 | -0.07 ( -0.01; -0.12) | 0.017 |
| **J01XA-Glycopeptides** | | | |  |  |  |  |
|  | 1.13 (1.10; 1.15) | 1.22 (1.19; 1.25) | 1.16 (1.13; 1.19) | 0.09 (0.13; 0.05) | <0.001 | -0.06 ( -0.02; -0.11) | <0.001 |
| **J01XB-Polymyxins (parenteral)** | | | |  |  |  |  |
|  | 0.029 (0.026; 0.031) | 0.022 (0.019; 0.026) | 0.015 (0.012; 0.018) | -0.006 (-0.002; -0.011) | 0.008 | -0.007 (-0.003; -0.012) | 0.005 |
| **J01XX01-Fosfomycin (parenteral)** | | | | |  |  |  |
|  | 0.47 (0.45; 0.48) | 0.64 (0.63; 0.66) | 0.64 (0.62; 0.65) | 0.18 (0.20; 0.16) | <0.001 | -0.01 (0.02; -0.03) | 0.505 |
|  |  |  |  |  |  |  |  |
| Table S6 | **Pre-pandemic phase** | **Pandemic phase** | **Transition phase** | **Difference** | | **Difference** | |
| continued | **2017-19** | **2020-21** | **2022-2023** | **pre-pandemic- pandemic** | | **pandemic-transition** | |
|  | **Mean Value (95%CI)** | **Mean Value (95%CI)** | **Mean Value (95%CI)** | **Difference (95%CI)** | **p-value** | **Difference (95%CI)** | **p-value** |
| **J01XX08-Linezolid** | | | |  |  |  |  |
|  | 0.71 (0.69; 0.74) | 0.88 (0.85; 0.91) | 0.85 (0.82; 0.88) | 0.17 (0.21; 0.13) | <0.001 | -0.03 (0.01; -0.08) | 0.169 |
| **J01XX09-Daptomycin** | | | | |  |  |  |
|  | 0.27 (0.25; 0.29) | 0.37 (0.34; 0.39) | 0.40 (0.37; 0.42) | 0.1 (0.13; 0.06) | <0.001 | 0.03 (0.07; -0.01) | 0.109 |
| **J01AA12-Tigecyclin** | | | |  |  |  |  |
|  | 0.129 (0.122; 0.136) | 0.126 (0.117; 0.135) | 0.122 (0.113; 0.131) | -0.003 (0.009; -0.015) | 0.581 | -0.004 (0.009; -0.017) | 0.526 |
| **J01DD52-Ceftazidim/avibactam** | | | |  |  |  |  |
|  | 0.011 (0.009; 0.014) | 0.028 (0.025; 0.032) | 0.029 (0.026; 0.032) | 0.017 (0.021; 0.013) | <0.001 | 0.001 (0.006; -0.004) | 0.755 |
| **J01DF-Monobactams** | |  |  |  |  |  |  |
|  | 0.001 (-0.000; 0.002) | 0.001 (0.000; 0.003) | 0.007 (0.006; 0.009) | 0.001 (0.002; -0.001) | 0.343 | 0.006 (0.008; 0.004) | <0.001 |
| **J01DI04-Cefiderocol** | | | |  |  |  |  |
|  | n.a. ^a^ | 0.003 (0.002; 0.004) | 0.049 (0.039; 0.058) | n.a. | n.a. | 0.029 (0.042; 0.015) | 0.003 |
| **J01DI54-Ceftolozan/Tazobactam** | | | |  |  |  |  |
|  | 0.0089 | n.a. | 0.011 (0.008; 0.014) | n.a. | n.a. | n.a. | n.a. |

^a^ n.a., not applicable
